# Prebiotic supplementation in frail older people affects specific gut microbiota taxa but not global diversity

**DOI:** 10.1186/s40168-019-0654-1

**Published:** 2019-03-13

**Authors:** Tam T. T. Tran, Fabien J. Cousin, Denise B. Lynch, Ravi Menon, Jennifer Brulc, Jillian R.-M. Brown, Eileen O’Herlihy, Ludovica F. Butto, Katie Power, Ian B. Jeffery, Eibhlís M. O’Connor, Paul W. O’Toole

**Affiliations:** 1 APC Microbiome Ireland, University College Cork, National University of Ireland, Cork, Ireland; 2 School of Microbiology, University College Cork, National University of Ireland, Cork, Ireland; 3 Digestive Health Research Institute and Division of Gastrointestinal UH, Case Western Reserve University School of Medicine, Cleveland, OH USA; 4 Department of Biological Sciences, University of Limerick, Limerick, Ireland; 5 Health Research Institute, University of Limerick, Limerick, Ireland; 6 Department of Medicine, University College Cork, National University of Ireland, Cork, Ireland; 7 The Bell Institute of Health and Nutrition, General Mills Inc., Minneapolis, MN USA

**Keywords:** Aging, Microbiome, Prebiotics, Gut microbiota, Elderly, Innate immune response

## Abstract

**Background:**

There are complex interactions between aging, frailty, diet, and the gut microbiota; modulation of the gut microbiota by diet could lead to healthier aging. The purpose of this study was to test the effect of diets differing in sugar, fat, and fiber content upon the gut microbiota of mice humanized with microbiota from healthy or frail older people. We also performed a 6-month dietary fiber supplementation in three human cohorts representing three distinct life-stages.

**Methods:**

Mice were colonized with human microbiota and then underwent an 8-week dietary intervention with either a high-fiber/low-fat diet typical of elderly community dwellers or a low-fiber/high-fat diet typical of long-stay residential care subjects. A cross-over design was used where the diets were switched after 4 weeks to the other diet type to identify responsive taxa and innate immunity changes. In the human intervention, the subjects supplemented their normal diet with a mix of five prebiotics (wheat dextrin, resistant starch, polydextrose, soluble corn fiber, and galactooligo-saccharide) at 10 g/day combined total, for healthy subjects and 20 g/day for frail subjects, or placebo (10 g/day maltodextrin) for 26 weeks. The gut microbiota was profiled and immune responses were assayed by T cell markers in mice, and serum cytokines in humans.

**Results:**

Humanized mice maintained gut microbiota types reflecting the respective healthy or frail human donor. Changes in abundance of specific taxa occurred with the diet switch. In mice with the community type microbiota, the observed differences reflected compositions previously associated with higher frailty. The dominance of *Prevotella* present initially in community inoculated mice was replaced by *Bacteroides*, *Alistipes*, and *Oscillibacter*. Frail type microbiota showed a differential effect on innate immune markers in both conventional and germ-free mice, but a moderate number of taxonomic changes occurring upon diet switch with an increase in abundance of *Parabacteroides*, *Blautia*, *Clostridium* cluster IV, and *Phascolarctobacterium*. In the human intervention, prebiotic supplementation did not drive any global changes in alpha- or beta-diversity, but the abundance of certain bacterial taxa, particularly *Ruminococcaceae* (*Clostridium* cluster IV), *Parabacteroides*, *Phascolarctobacterium*, increased, and levels of the chemokine CXCL11 were significantly lower in the frail elderly group, but increased during the wash-out period.

**Conclusions:**

Switching to a nutritionally poorer diet has a profound effect on the microbiota in mouse models, with changes in the gut microbiota from healthy donors reflecting previously observed differences between elderly frail and non-frail individuals. However, the frailty-associated gut microbiota did not reciprocally switch to a younger healthy-subject like state, and supplementation with prebiotics was associated with fewer detected effects in humans than diet adjustment in animal models.

**Electronic supplementary material:**

The online version of this article (10.1186/s40168-019-0654-1) contains supplementary material, which is available to authorized users.

## Background

Diet has an important role in shaping gut microbiota composition and function. Numerous studies have reported an association between consumption of non-digestible fiber ingredients (e.g., prebiotics) and increased levels of reputedly beneficial bacteria in the gut such as *Ruminococcaceae*, *Bifidobacterium*, *Lactobacillus*, *Faecalibacterium*, and *Roseburia* [1–4]. An overview of 2099 participants from 64 studies revealed a significant increase in the abundance of bacteria from the genera *Bifidobacterium* and *Lactobacillus* as well as higher levels of fecal butyrate concentration following dietary fiber intervention, particularly involving fructans and galacto-oligosaccharides, compared with placebo/low-fiber comparators [5]. Similarly, the consumption of resistant starch has been shown to enrich *Ruminococcus bromii* and *Eubacterium rectale* [6]. The gut microbiota interacts with the human immune system and microbiota alterations have been linked to various inflammatory, metabolic, or functional disorders such as type 2 diabetes, obesity, and irritable bowel syndrome [7–9]. One of the mechanisms implicated in immune modulation is gut microbiota fermentation of dietary fibers to produce short-chain fatty acids (SCFAs) [10]. Studies of the Mediterranean diet showed that a diet rich in fruit, legumes, and vegetables affected the abundance of certain gut bacteria, increased levels of fecal SCFAs [11], and reduced inflammation in Crohn’s disease [12]. In contrast, a low-carbohydrate diet was associated with increased abundance of *Streptococcus*, *Lactococcus*, and *Eggerthella*, and lower abundance of carbohydrate-degrading bacteria (*Ruminoccocus*, *Eubacterium*, *Clostridium*, and *Bifidobacterium*), resulting in reduction in fecal concentrations of SCFAs [13]. Moreover, a progressive loss of microbial diversity was observed in mice colonized with a human microbiota consuming a low-fiber diet over generations, which was not recoverable after the reintroduction of dietary fiber [14].

Changes in the gut microbiome are also associated with aging [15–17]. In our previous studies, we described the relationship between aging, frailty, diet, and the gut microbiota. Microbiota composition was a strong co-variate of measures of frailty, comorbidity, nutritional status, and markers of inflammation [15, 16]. A reduction in abundance of particular taxa such as *Ruminococcus* and *Prevotella* in the elderly, with an associated increase in *Alistipes* and *Oscillibacter*, correlated with several indices of frailty, and we reported a direct correlation between values of the healthy food diversity (HFD) index (dietary diversity as well as amount and health-promoting value of consumed food [18]) and gut microbiota alpha diversity (gut microbiota richness within individuals) in both community and long-stay subjects [15, 17]. Levels of inflammatory markers including TNF-α, IL-6, and IL-8 and C-reactive protein (CRP) are higher in long-stay and rehabilitation subjects than in community dwellers [15]. Moreover, as hypothesized frequently and summarized in a recent review [19], the gut microbiota may play a key role in age-related inflammation.

Based on the evidence reviewed above, gut microbiota modulation by diet is a potential approach for extending health span in the elderly. In this study, we investigated whether gut microbiota transfer from older, healthy community-dwelling, and long-term residential care individuals could be stably established and maintained in mice fed a customized diet; whether these microbiota compositions would respond to dietary modification; and if there was an effect upon innate immunity markers. In addition, a dietary intervention in elderly subjects and young-healthy controls was conducted to determine if prebiotic supplementation could modulate gut microbiota composition and reduce inflammation in the elderly.

## Results

### Establishment and dietary programming of elderly donor gut microbiota in mice

**Fig. 1:**
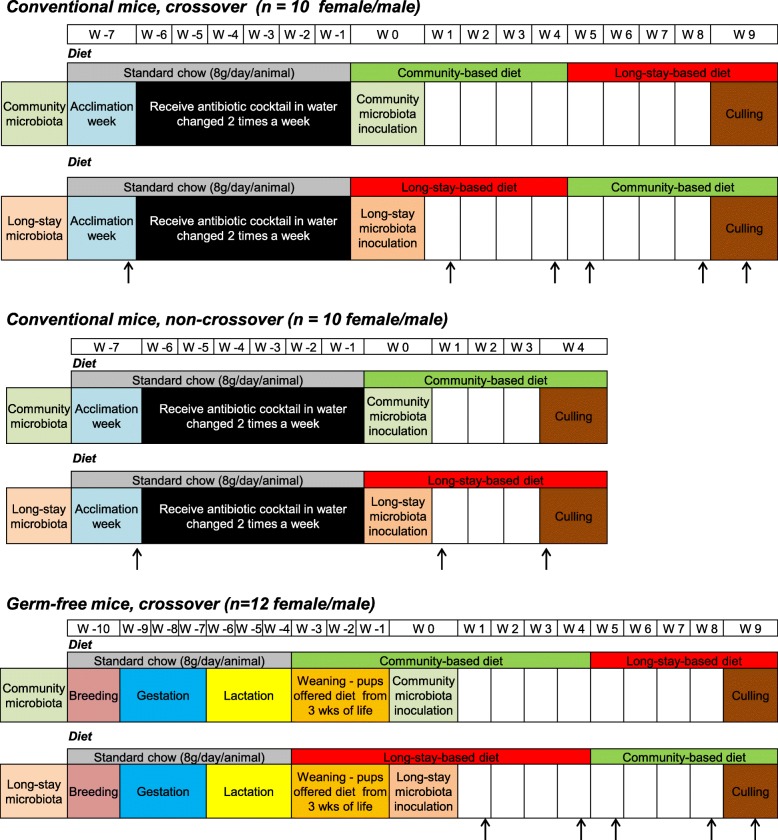
Schematic diagram of experimental design in murine intervention study. Arrows indicate the week on which the fecal samples were collected

Conventional mice that had been treated with a cocktail of five antibiotics (ampicillin, vancomycin, ciprofloxacin, imipenem, metronidazole) for 6 weeks, and germ-free mice, were humanized with cryo-preserved fecal material (see “Methods” section) from frail (long-stay) or healthy (community dwelling) elderly donors. The fecal microbiota was sampled at critically informative time-points, namely just before antibiotic treatment, in the week following humanization, after 4 weeks on the first experimental diet, in the week after diet swap (week 5), and at the end of the second diet period (week 8) (Fig. 1**)**. To examine diet-microbiome interaction in mice of human microbiota corresponding to different older health strata, we commissioned the production of two synthetic diets (Table 1). The main differences between the two diets was a higher proportion of sugars, meat-derived ingredients, and fats that provide 3.6 kcal/g in the long-stay-based diet, whereas higher amounts of starches, fibers, and fish products provide 4.5 kcal/g in the community-based diet, which contained an additional 284 g/kg of dietary fiber and 38 g/kg cellulose. The mice were allowed to feed ad libitum from 8 g fresh feed provided daily.

**Table 1:** Composition of community-based diet and long-stay-based diet

	Community-based diet	Long-stay-based diet
	g/kg	g/kg
Ingredients		
Sucrose	160	330
Corn starch	62.7	101.5
Maltodextrin	50	90
High amylose corn starch	150	0
RS3-resistant starch^a^	100	0
Cellulose	43	5.2
Soluble corn fiber^b^	36	1.5
l-cystine	3	3
Casein	63	0
Fish meal, menhaden	88.5	0
Meat and bone meal (50%)	0	85
Poultry meal, low ash (11%)	81	160
Soybean oil	25	15
Lard	60	96
Coconut oil, hydrogenated	20	55
Mineral Mix, AIN-93G-MX	40	40
Vitamin Mix, AIN-93-VX	15	15
Choline bitartrate	2.75	2.75
Thiamin HCl	0.013	0.013
Vitamin K_1_, phylloquinone	0.002	0.002
TBHQ, antioxidant	0.014	0.014
Selected nutrient (% kcal)		
Protein	18.5	13.9
Carbohydrate	50.7	47.1
Fat	30.8	38.9

*RS3* resistance starch type 3, *TBHQ* tert-butylhydroquinone

^b^Promitor soluble fiber (Tate & Lyle Lafayette, Indiana, USA)

^a^Novelose 330 (Ingredion, Indianapolis, Indiana, USA)

**Fig. 2:**
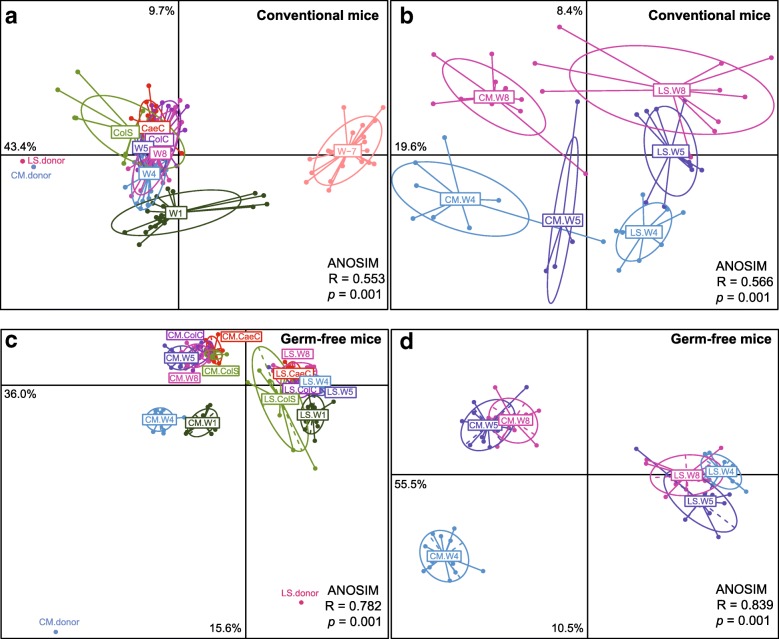
Principle coordinates analysis plots based on Spearman distances colored by time point. Samples from conventional mice cross over design: **a** all samples; **b** week 4, 5, and 8 samples. Samples from germ-free mice cross over design: **c** all samples, **d** week 4, 5 and 8 samples. The significant differences between groups were calculated by analysis of similarity (ANOSIM) tests. *CM donor* healthy community subject, *LS donor* frail long-stay subject, *ColS* colonic scraping, *ColC* colonic content, *CaeC* caecal content, *W* week, *W*-*7* (week minus 7) murine microbiota before antibiotic treatment

Beta diversity (PCoA) analysis showed that the microbiota of the pre-treatment conventional mice was very distinct from that of the human donors whose datasets grouped together on this phylogenetic distance scale (Fig. 2a). However, when sampled 1 week after inoculation, the microbiota of the humanized mice was closer to that of the microbiota of the human donors and remained stable for 4 weeks on the diet emulating the healthy human diet. On the scale where all samples are mapped (Fig. 2a), it is not possible to see changes between later weeks, but nevertheless it is clear that the microbiota of the colonic scrapings, colonic contents, and caecal contents at week 8 are close to that of the fecal microbiota at week 8. Even though the bacterial community composition differed among these groups at week 8, the microbial communities from the feces and colonic contents are more similar to each other than are the colonic scrapings and caecal contents in both conventional mice and germ-free mice (Additional file 1: Figure S1). These findings confirm that the fecal analysis is a good proxy for luminal gut microbiota analysis in this context.

Fine-scale PCoA analysis (Fig. 2b) revealed that the microbiota of mice colonized with material from a long-stay donor, as expected, was separate from that of mice that received fecal microbiota from a health community donor. After 4 weeks, the murine diets were swapped. After only 1 week (i.e., by week 5) on the high-fat low-fiber long-stay type diet, the community-colonized mouse microbiota moved noticeably in the PCoA toward that of the long-stay colonized mice. The microbiota of the long-stay colonized mice switched to a “healthy” diet moved away from the community quadrant after 1 week, and over half the samples continued in that direction from weeks 5 to 8, while a minority converged toward the community-mice on the low-fiber high-fat long-stay diet. Between weeks 5 and 8, the community-colonized mice fed a low-fiber high-fat long-stay-based diet moved back into their original side of the PCoA plot but did not revert to their original position.

Microbiota composition analysis and identification of differentially abundant taxa between time points revealed that antibiotic treatment and inoculation led to successful engraftment of the human microbiota. Moreover, genus abundance changes were associated with diet change. Community-inoculated mice that were transferred to the low-fiber high-fat long-stay-based diet showed an increase in the relative abundance of *Bacteroides*, *Alistipes*, *Pseudoflavonifractor*, *Oscillibacter*, *Clostridium* cluster XI, *Desulfovibrio*, and *Butyricimonas* and a decrease in *Prevotella* abundance (Additional file 1: Figure S2a). Long-stay inoculated mice showed an increase in abundance of *Parabacteroides* and *Marvinbryantia* and a decrease in *Prevotella*, *Flavonifractor*, *Oscillibacter*, *Clostridium* cluster XI, *Desulfovibrio*, and *Butyricimonas* when switched to the high-fiber low-fat community-based diet (Additional file 1: Figure S2b).

Similar analyses were performed in germ-free mice that had been colonized, their microbiota monitored for 4 weeks, and then subjected to dietary swap. Microbiota composition was initially distinct, reflecting that of the two human microbiota donor types, but switched based on the change of diet (Additional file 1: Figure S3). Similar to the conventional animals, the low-fiber high-fat long stay-based diet switch in the animals inoculated with community-type microbiota had a greater impact on the gut microbiota than did the change to the community-based diet in long-stay colonized animals (Fig. 2c, d). Thus, the long-stay type microbiota was relatively non-responsive to the introduction of the high-fiber low-fat community-based diet. However, specific responses (Additional file 1: Figure S3b) included increased proportions of the health-associated taxa *Blautia* and *Clostridium* cluster IV and decreased relative abundance of *Parasutterella* (which is associated with induced colitis [20]) and *Bacteroides*.

In order to compare success of engraftment of the human microbiota in the two mouse models, we examined the datasets at week 1 after inoculation and compared the number of observed genera with those in the inoculating donor stool (Additional file 2: Table S1). Of the 54 genera observed in the stool of the long-stay donor, 46 were detected in at least 1 germ-free mouse, whereas 45 genera were detected in at least 1 antibiotic-treated conventional mouse. We then set a threshold that at least 50% of the recipient mice must harbor the genus to be considered successful engraftment. By this criterion, 36 genera were successfully engrafted in the germ-free mice, whereas only 26 were successfully introduced into the antibiotic-treated conventional mice. Six genera were found in germ-free mouse stool that were not present in the donor stool, and four of those were found in 50% of the mice, presumably acquired from the environment post humanization. However, 26 genera were found in antibiotic-treated conventional mice that were not found in the donor stool, although only 1 of these was found in a minimum of 50% of the mice. Applying a similar analysis to the community donor inoculum, of the 46 genera observed in the community donor inoculum, 37 were successfully engrafted into any antibiotic-treated conventional mice but only 33 were engrafted into the germ-free mice. Of those, 22 were engrafted into 50% of the antibiotic-treated conventional mice, although 27 were engrafted into 50% of the germ-free mice. Twenty-one genera were found in the antibiotic-treated conventional mice stools that were not found in the donor stool, 4 of which were found in 50% of mice, whereas 18 genera were found in the germ-free mice that were not found in the donor, 10 of which were found in 50% of mice. More generally, while similar numbers of genera engraft in any type of mouse, engraftment appears successful in a higher proportion of germ-free mice than conventional mice. Conventional mice were also carrying higher numbers of non-transferred genera, presumably having survived antibiotic treatment.

### Differential effects of healthy and frail microbiota types on innate immunity in mice

**Fig. 3:**
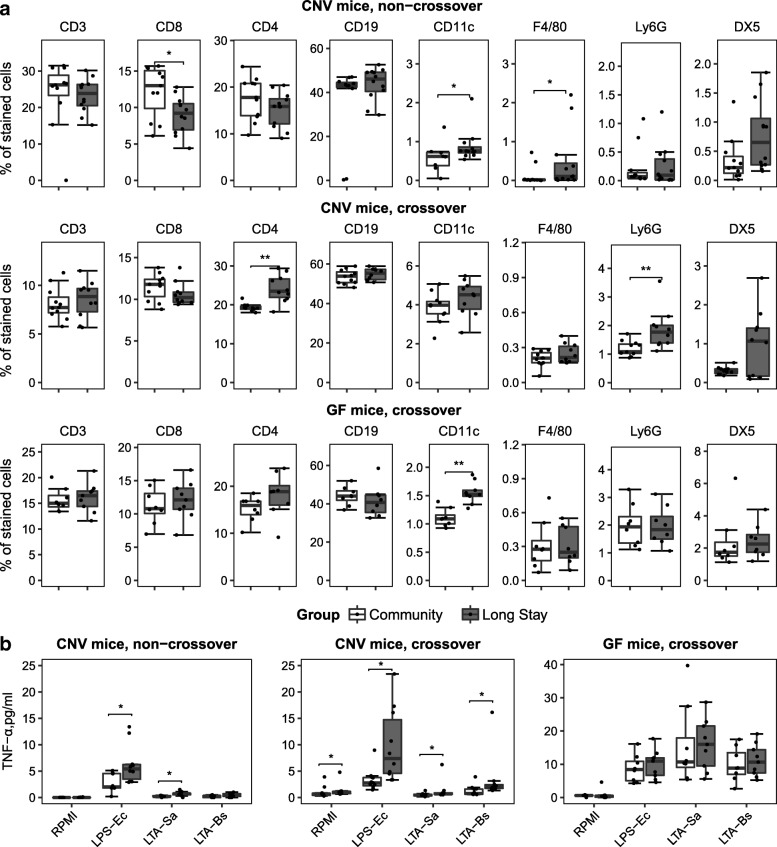
Splenocyte phenotypes and TNF-α response profiles of mice inoculated with a community microbiota and mice inoculated with a long-stay microbiota in conventional mice (CNV) and germ-free (GF) mice. **a** Immunostaining of splenocytes. **b** TNF-α response after splenocyte stimulation with RPMI complete medium as control (RPMI) or with bacterial surface compounds including LPS from *Escherichia coli* K12 (LPS-Ec), LTA from *Staphylococcus aureus* (LTA-Sa) or from *Bacillus subtilis* (LTA-Bs). *P* values were calculated using the Mann-Whitney *U* test, *p < 0.05; **p < 0.001

Next, we investigated the differential effect of introducing the two elderly subject microbiota types on the murine innate immune system. Spleen mononuclear cells isolated from long-stay inoculated mice in both the crossover design and non-crossover design experiments showed higher level expression of many T cell markers associated with inflammation, compared to mice receiving fecal inoculum from a healthy community dwelling subject. In particular, the long-stay inoculated conventional mice, non-crossover design, presented higher proportions of CD11c-expressing dendritic cells and F4/80-expressing macrophages, and lower proportions of CD8^+^ T cells. However, in the crossover design, we observed an increased abundance of CD4^+^ T regulatory cells and Ly6G^+^ neutrophils in the long-stay inoculated conventional mice. An increased proportion of CD11c expressing cells was also noted in the long-stay inoculated germ-free mice (Fig. 3a). After splenocyte stimulation, the long-stay inoculated conventional mice had higher TNF-α levels in the supernatant than the community-microbiota inoculated mice. We also detected higher TNF-α expression levels after LPS-K12 and LTA-SA stimulation in the long-stay type microbiota-inoculated mice compared to community inoculated mice in the conventional mouse non-crossover design. Similar observations were found for TNF-α level after stimulation and even in RPMI control in the conventional mouse crossover design. No difference in TNF-α levels between diet-microbiota treatment groups was observed and a very high level of responses to all three stimuli was measured in germ-free mice (Fig. 3b).

### Design and implementation of a prebiotic supplementation trial in human subjects

Prebiotic compounds which can be metabolized by a subset of intestinal bacteria are a recognized way of modulating the microbiota [21]. Since the microbiota of frail long-stay subjects displays loss of certain taxa, it might be possible to “remediate” this by prebiotic supplementation. We surveyed the literature then available for microbiota effects of prebiotics, focusing mainly on trials in older humans (Table 2), ingredient type, and tolerated dosage, cognizant of the challenge of introducing prebiotics in the diet of the elderly [22]. Collectively, these compounds had been shown in previous studies (Table 2) to promote the growth of a diverse range of health associated taxa, including those depleted in the low-diversity microbiota of frail older people. We thus designed a daily supplement containing five prebiotic ingredients of which almost half were resistant starch, an ingredient known to promote growth of health-related taxa in the gut microbiota [6]. The placebo treatment was 10.5 g/day of maltodextrin, previously shown to have relatively low effect of the gut microbiota [23]. As well as comparison to maltodextrin effects, each subject acted as their own control because the main focus was microbiota comparison pre- and post-intervention. After a 2-week lead-in, the trial was conducted for 6 months (26 weeks) with a 6-week washout after which further microbiota analysis was performed.

**Table 2:** Selection, source, and dosage of prebiotic ingredients for dietary supplementation

Ingredient [Reference]	Trial	Outcomes	Supplier/product code	g/mix
Wheat dextrin [43]	Intensive care unit patients (~ 65 years) 10–22 g/day × 2–5 weeks	Increase in *Firmicutes*, butyrate-producing bacteria (*Eubacterium hallii*, *Eubacterium rectale*, *Roseburia intestinalis*, *Clostridia cluster XIVa*), starch degrading bacteria (*Ruminococcus bromii*, *Ruminococcus obeum*, *Sporobacter terminitidis*), *Fecalibacterium prausnitzii*, *Bifidobacterium* spp., & SCFA levels	Ingredion/Nutriose FB 06	3
Resistant starch [24]	Rats 10%–20 weeks	Reduced incidence of colon carcinogenesis possibly through an increase in butyrate	Ingredion/Novelose 330	10
Polydextrose [3]	Healthy males (20–40 years) 21 g/day × 21 days	Increase in *Faecalibacterium*, *Phascolarctobacterium* and *Dialister*	Danisco/Litesse	3
Soluble corn fiber [3]	Healthy males (20–40 years) 21 g/day × 21 days	Increase in *Faecalibacterium*, *Phascolarctobacterium*, *Dialister*, *Roseburia* and *Lactobacillus*	Tate and Lyle/ SCF 22–5421/Promitor soluble fiber 85	3
Galactooligo-saccharide [4]	Healthy humans (19–50 years) 5–10 g/day × 16 weeks	Bifidogenic, increase in *Fecalibacterium prausnitzii* and *Actinobacteria* and reduction in *Bacteroides*	Friesland Campina/Vivinal GOS	2
Total max daily supplement				21

**Fig. 4:**
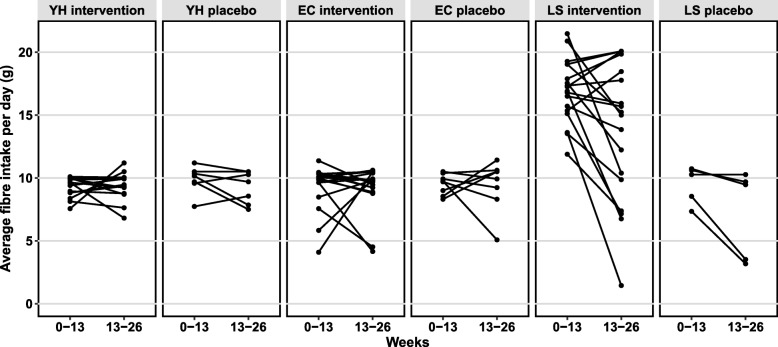
Average intake of dietary supplement per day as an intervention or placebo across subjects from week 0 to week 13, and week 13 to week 26. *YH* young-healthy, *CM* community, *LS* long-stay

To provide additional comparative value, we recruited three treatment groups: young-healthy subjects, elderly community subjects (a healthy elderly group), and elderly long-stay subjects, descriptive statistics for which are shown in Table 3. We aimed to have 20 treated subjects and 10 placebo controls in each of the 3 arms. This recruitment ratio of 2 to 1 was designed to provide the optimal number of samples so that alterations due to the treatment could be detected and characterized, while also allowing us to recruit and sample a placebo group. However, only 69 subjects out of 93 recruited participants (74.1%) completed the study, due to a combination of infraction of exclusion criteria; death due to unrelated causes (5.4%, *n* = 5); withdrawal at the investigators’ discretion, usually because of deteriorating health (5.4%, n = 5); self-withdrawal due to unspecified personal reasons (9.7%, *n* = 9); loss to follow up (2.1%, *n* = 2); or inability to produce samples (3.2%, *n* = 3) (Additional file 2: Table S2). As expected, the three cohorts showed significantly different clinical status, especially the young-healthy/community and long-stay with respect to age, frailty (Charlson comorbidity index, Barthel index), HFD value, and other clinical markers except BMI and sex (Table 3). However, within each cohort, there was no significant difference between intervention and placebo arms at baseline except sex ratio and HFD index in the young-healthy group due to skewed withdrawal of females from the placebo group (Table 3). Compliance/dosage received varied widely especially in long-stay subjects (Fig. 4). Rather than losing participants from the study, tolerance was monitored weekly, and after the first 13-week period, the daily prebiotic intake was reduced if required in long-stay intervention subjects. 16S rRNA gene amplicon sequencing was used to profile the microbiota of available subjects/samples at each time point (see Additional file 2: Table S3).

**Table 3:** Clinical characteristics of the subjects, grouped by residence location, at baseline

Characteristics	Young-healthy			Community			Long-stay			*p* ^b^
	Intervention	Placebo	*p* ^a^	Intervention	Placebo	*p* ^a^	Intervention	Placebo	*p* ^a^	
*n*	21	8		20	8		17	5		
Sex (#male:female)	6:15	7:1	0.01	7:13	3:5	1.00	7:10	2:3	1.00	0.808
Age (years)	41.9 (14.5)	33.1 (13.2)	0.09	69.5 (4.9)	74.9 (7.5)	0.07	83.4 (7.6)	85 (7.6)	0.84	< 0.001
BMI (kg/cm^2^)	24.7 (5.0)	25.7 (3.5)	0.31	26.2 (4.3)	25.5 (1.4)	0.76	27.3 (6.2)	28.8 (7.7)	0.82	0.157
Weight (kg)	70.6 (13.5)	80.4 (10.6)	0.06	74.7 (14.9)	66.2 (8.8)	0.13	80 (28.1)	90.7 (28.5)	0.46	0.665
Charlson comorbidity	ND	ND	ND	0 (0)	0.1 (0.4)	0.13	2 (1.3)	3 (3.0)	0.75	< 0.001
Barthel score	ND	ND	ND	20 (0)	19.9 (0.4)	0.13	6.6 (5.5)	5 (3.5)	0.72	< 0.001
ALP (IU/L)	73.8 (26.0)	90.5 (24.6)	0.10	80.8 (16.6)	74.4 (17.7)	0.41	106.2 (28.9)	101.5 (39.3)	0.79	0.002
ALT (IU/L)	25.1 (12.6)	26.5 (5.9)	0.29	23.8 (10.9)	24 (7.7)	0.58	13.9 (7.2)	12.5 (4.5)	0.86	< 0.001
AST (IU/L)	25.2 (4.5)	28.8 (6.8)	0.19	27.3 (7.1)	31.2 (13.6)	0.94	19 (4.9)	16 (3.2)	0.33	< 0.001
Bilirubin (μmol/L)	14.1 (10.6)	10.9 (5.0)	0.45	12.1 (5.8)	11.1 (4.5)	0.75	6.5 (2.0)	7 (2.4)	0.65	< 0.001
Total cholesterol (mmol/L)	5.5 (0.7)	5.7 (1.1)	0.90	5.5 (1.2)	5.1 (1.2)	0.49	4.5 (0.9)	4.8 (1.9)	0.87	0.004
HDL-cholesterol (mmol/L)	1.5 (0.3)	1.4 (0.3)	0.15	1.6 (0.3)	1.8 (1.2)	0.30	1.2 (0.3)	1.1 (0.2)	0.64	0.001
LDL-cholesterol (mmol/L)	3.4 (0.6)	3.6 (0.9)	0.59	3.5 (1.2)	2.9 (0.8)	0.29	2.6 (0.8)	2 (0.8)	0.40	0.004
Triglycerides (mmol/L)	1.3 (0.5)	1.5 (0.6)	0.59	1.2 (0.4)	1.3 (0.3)	0.33	1.7 (0.8)	2.8 (1.9)	0.23	0.030
Fasting glucose (mmol/L)	4.5 (0.6)	4.5 (0.5)	0.48	5.2 (0.7)	5 (0.6)	0.49	6.1 (1.6)	7.7 (2.8)	0.22	< 0.001
Creatinine (μmol/L)	71.5 (12.2)	80.9 (15.8)	0.07	70.1 (14.4)	76.1 (23.3)	0.52	91 (48.0)	104.5 (38.9)	0.59	0.068
Ferritin (μg/L)	73.8 (48.5)	98.1 (64.5)	0.39	102.6 (63.2)	107.6 (53.7)	0.61	80.9 (70.7)	40.3 (10.1)	0.79	0.094
TSH (mIU/L)	1.9 (0.8)	1.6 (0.3)	0.39	2.5 (1.3)	2.4 (1.3)	0.92	1.7 (0.6)	1.1 (1.0)	0.30	0.037
Urea (mmol/L)	5.4 (1.1)	5.5 (1.2)	0.96	5.7 (1.4)	5.5 (1.0)	0.58	8.7 (3.6)	11.6 (4.1)	0.11	< 0.001
Vitamin B_12_ (pg/mL)	265.1 (159.3)	318.8 (120.6)	0.12	332.6 (153.3)	303.4 (93.4)	0.79	234.2 (119.5)	195.7 (46.6)	0.63	0.009
White blood cell count	6 (1.6)	6.1 (1.4)	0.79	5.5 (1.1)	6.2 (1.7)	0.47	7.3 (2.6)	6.9 (0.7)	0.90	0.042
HFD index	0.5 (0.1)	0.4 (0.1)	0.031	0.4 (0.1)	0.4 (0.1)	0.409	0.3 (0.1)	0.4 (0.1)	0.179	< 0.001

Data presented as mean (SD)

*ALP* alkaline phosphatase, *ALT* alanine aminotransferase, *AST* aspartate transaminase, *TSH* thyroid-stimulating hormone, *HFD* healthy food diversity, *ND* not done

^a^Mann-Whitney *U* test for two treatment groups except Fisher’s test for sex ratio

^b^Kruskal-Wallis test for three residence location groups

### Limited effect of prebiotic supplementation on gut microbiota

**Fig. 5:**
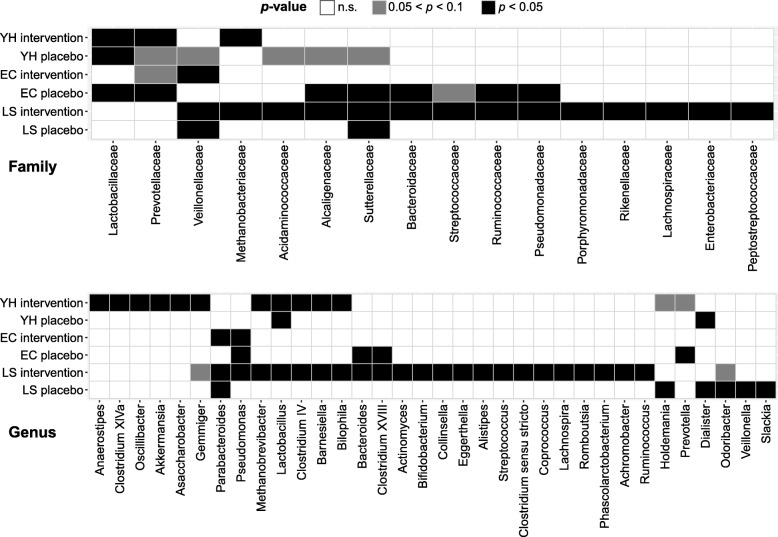
Significantly differential abundant taxa at family and genus level in any time points within treatment groups. Adjusted *p* values were calculated by DESeq2 test with Benjamini–Hochberg correction. *YH* young-healthy, *CM* community, *LS* long-stay

The microbiota alpha diversity of long-stay subjects was significantly lower than that in community and young-healthy subjects, but there were no significant differences in alpha diversity within residence location for treatment groups at baseline except in Chao1, community group (Additional file 1: Figure S4**)**. Bray-Curtis distance PCoA showed that fecal microbial profiles of long-stay subjects were separated significantly from those of community and young-healthy at baseline, in line with our previous studies [15] (Additional file 1: Figure S5). No statistically significant difference in alpha- and beta-diversity was found between time points within treatment groups (Additional file 1: Figures S6, S7). Furthermore, there was no significant interaction between the dosage received and alpha diversity within each intervention treatment groups (Additional file 2: Table S4), suggesting that an overall treatment effect on microbiota diversity was not being masked by the range of prebiotic dosage received. Variations in the relative abundances of numerous fecal microbiota taxa were detected in individuals over time points and between individuals (Additional file 1: Figure S8). Analysis using DESeq2 identified 15 families and 34 genera that were differentially abundant between time points in treatment groups (Fig. 5**,** Additional file 3: Tables S5, S6), without a clear treatment effect for most. However, among these, several taxa were commonly associated with intervention or placebo treatment. Notably, increased abundance was observed for some bacteria after prebiotic supplementation such as *Ruminococcaceae* (*Clostridium* cluster IV), *Parabacteroides*, and *Phascolarctobacterium* (Additional file 1: Figure S9). In addition, to examine the common signature associated with the prebiotic and placebo interventions, we conducted an analysis on all available intervention or placebo samples regardless of life-stage. Seven taxa representing one unclassified *Ruminococcaceae* and six genera were significantly differentially abundant across all placebo-treated subjects, but were not significantly different in the individual cohorts except *Pseudomonas* (Additional file 1: Figure S10). In contrast, 11 taxa representing 2 unclassified and 8 genera were significantly altered in abundance in the prebiotic intervention subjects. There taxa were consistently returned between the individual cohorts and the combined analysis with all identified genera being differentially abundant in at least one of the life-stage intervention cohorts.

### Immunological/inflammatory biomarker changes associated with prebiotic supplementation

Persistent inflammation is associated with less healthy aging and was a target for reduction by dietary modulation of the gut microbiota. Levels of cytokines and inflammatory biomarkers (CRP, CXCL11, CCL11) were higher at baseline in long-stay subjects than in community and young-healthy subjects (Table 4). Levels of IL-17A, IL6, TNF-α, and lipopolysaccharide (LPS) were higher in community dwelling elderly participants than in young-healthy subjects (recognizing the limitation that the reporter cell assay for LPS was sensitive to other molecules that might trigger NFkB activation). There was no significant difference between groups at baseline in proportion of CD4^+^ cells, while CD8^+^ levels were higher in young-healthy subjects compared to others.

**Table 4:** Comparison of immunological/inflammatory biomarkers between residence location groups at baseline

Biomarkers	*p* ^a^	*p* ^b^			Mean (SD)		
		CM vs. YH	CM vs. LS	YH vs. LS	YH	CM	LS
CRP (mg/L)	< 0.001	0.443	< 0.001 (↑)	< 0.001 (↑)	2 (3.8)	1.5 (1.2)	24.3 (29.2)
CXCL11 (pg/ml)	< 0.001	0.244	< 0.001 (↑)	< 0.001 (↑)	345.8 (594.6)	670.3 (1310.9)	1278.1 (1655.1)
CCL11 (pg/ml)	< 0.001	0.126	< 0.001 (↑)	< 0.001 (↑)	12.1 (27.3)	17.5 (37.3)	310.3 (133.3)
IL-17A (pg/ml)	< 0.001	< 0.001 (↓)	0.002 (↑)	< 0.001 (↑)	0.4 (0.0)	0.9 (0.5)	3.4 (2.9)
IFN-γ (pg/ml)	0.004	0.094	0.026 (↑)	< 0.001 (↑)	5 (6.4)	6.4 (6.5)	10.5 (8.4)
IL-10 (pg/ml)	< 0.001	0.365	< 0.001 (↑)	< 0.001 (↑)	0.1 (0.1)	0.1 (0.1)	0.8 (1.9)
IL-6 (pg/ml)	< 0.001	0.006 (↓)	< 0.001 (↑)	< 0.001 (↑)	0.2 (0.1)	0.6 (0.4)	3.1 (3)
IL-8 (pg/ml)	0.008	0.113	0.033 (↑)	< 0.001 (↑)	8.9 (5.1)	12.9 (14.2)	29.1 (67.4)
TNF-α (pg/ml)	< 0.001	0.012	< 0.001 (↑)	< 0.001 (↑)	1 (0.6)	1.7 (0.8)	5.1 (2.8)
CD4 (%)	0.342	0.113	0.097	0.448	38.6 (7.8)	33.5 (13)	37.8 (16.3)
CD8 (%)	0.001	< 0.001 (↑)	0.167	0.006 (↓)	20.9 (6.6)	13.6 (9.5)	16.5 (10.6)
LPS (EU/ml)	0.065	0.010 (↓)	0.129	0.173	6.1 (4.7)	11 (8.3)	8.2 (7.0)

↑ ↓ Indicates direction of difference in value in the latter-named group, in the pairwise comparison

*CM* community (seniors), *YH* young-healthy, *LS* long-stay (residential care seniors)

^a^Kruskal-Wallis test across the three residence location groups

^b^Dunn’s post-hoc test for pairwise comparison

**Fig. 6:**
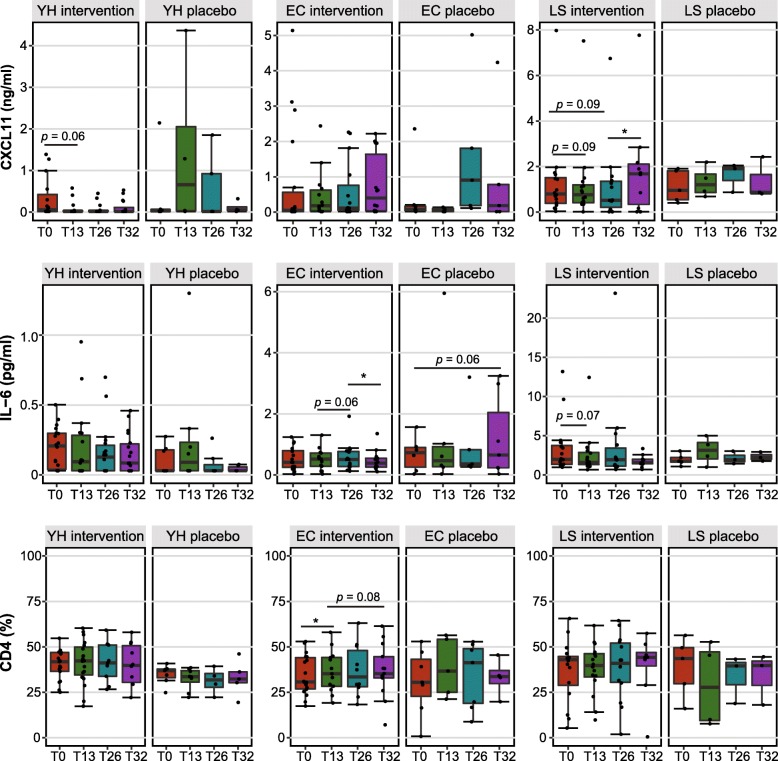
Serum CXCL11 and IL-6 levels, and CD4^+^ T cell proportions were different between time points within treatment groups. *P* values were calculated from Wilcoxon signed-rank test. *p < 0.05. *YH* young-healthy, *CM* community, *LS* long-stay, *T0* baseline (week 0), *T13* halfway (week 13), *T26* end of study (week 26), *T32* 6 weeks after the cessation of treatment (week 32)

Within treatment groups, there was a significant difference between time points in levels of CXCL11, CCL11, IL-6, IL-8, TNF-α, CD8, and LPS (Additional file 3: Table S7). Decreased plasma CXCL11 level tended to be associated with intervention treatment while the opposite pertained for IL-6 and CD4^+^ measures (Fig. 6). CXCL11 levels in supplemented long-stay subjects increased during the washout period, consistent with a treatment reduction effect. Decreased plasma CCL1 levels and CD8^+^ counts were positively associated with prebiotic supplementation in young-healthy subjects. Measures of IL-8, TNF-α, and LPS differed significantly between time points in both intervention and placebo subjects (Additional file 1: Figure S11).

## Discussion

Our previous studies suggested that programming of the gut microbiota by habitual diet might contribute to microbiota changes associated either causally or consequentially with loss of health in older people. In this study, we sought to clarify these possibilities by microbiota transfer to mice in which we could dramatically manipulate the diet, and in humans where more modest dietary alteration could be performed. Our data indicate that dietary programming is possible, confirming other studies in animals and humans [3, 24], although not as responsive in our pre-clinical models as in published studies of humans [25, 26]. In humans, the prebiotic supplementation had relatively modest effects on the microbiota and achieved some alteration of innate immune markers that may be beneficial in the elderly. This effect could be direct, i.e., not microbiota-mediated, by mechanisms including the prebiotic compounds binding to host metabolites, or being absorbed directly into intestinal cells and changing the expression level of cytokine genes [27].

**Fig. 7:**
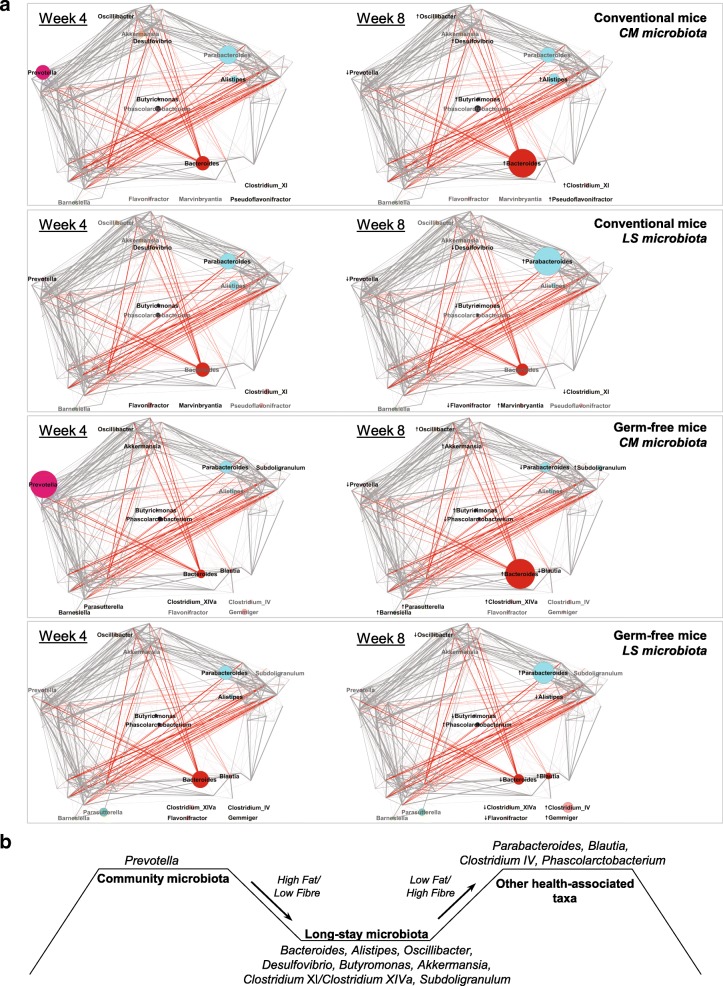
**a** Composition changes in gut microbiota at the genus level of conventional mice and germ-free mice inoculated with a community microbiota or with a long-stay microbiota were associated with dietary change. Genera were projected on to a pre-existing network of co-abundance groups as defined by Claesson et al. [15]. Circle sizes indicate genus abundance with arrow (↑ or ↓) indicating increases or decreases in taxon abundance in week 8 when compared to week 4. Associations between genera are represented by gray lines for positive and red lines for negative. **b** Schematic summary of how selected changes in gut microbiota composition are associated with fiber supplementation

To search for coherent signals between the current study and our previous analyses of microbiota in the elderly, taxa whose abundance changed significantly after dietary swap in mice were plotted on to a network of bacterial Co-abundance groups (CAGs) that we previously generated [15] (Fig. 7a) from the ELDERMET cohort. This data visualization graphically highlights the fact that the dominant genera associated with the CAGs in the original human cohort changed in abundance in the humanized mice after the diet swap in a way that was consistent with the compositional differences between healthy community-dwelling subjects’ microbiota and the long-stay type microbiota in the ELDERMET cohort. These transitions were associated with the reduction of fiber in both conventional and germ-free mice humanized with microbiota from healthy community-dwelling subjects. After dietary modification, the dominance of *Prevotella* presented initially in community inoculated mice was replaced by *Bacteroides*, *Alistipes*, and *Oscillibacter.* Although we observed decline of *Bacteroides*, *Alistipes*, and *Oscillibacter* in long-stay inoculated mice fed with the high-fiber/low-fat diet, the gut microbiota did not revert to a *Prevotella*-dominated configuration. Long-stay type taxa were instead replaced by other taxa including *Parabacteroides*, *Blautia*, *Clostridium* cluster IV, and *Phascolarctobacterium* (Fig. 7). Most taxonomic changes could be explained by ingredients associated with the diet types. The *Bacteroides* enterotype was strongly associated with protein and animal fat intake, while the *Prevotella* enterotype was associated with carbohydrates and simple sugars [25]. None of the fiber ingredients in the community-based diet that we used here has previously been reported to promote *Prevotella*, although the dominance of this genus, which was evident in community dwellers, might possibly be achieved by a long-term experimental diet regime. *Parabacteroides* and *Phascolarctobacterium* were positively associated with soluble corn fiber intake [3, 28], and both became more abundant in both murine and human intervention studies with soluble corn fiber supplementation. We also found that *Oscillibacter*, *Clostridium* cluster XI, *Desulfovibrio*, and *Butyricimonas* were more abundant in mice receiving the low-fiber/high-fat diet and decreased in abundance after a high-fiber/low-fat diet, largely consistent with previous murine studies [29–31]. A limitation of the current mouse study is that the inoculum for each dietary group was provided by one donor. Given the continuum of inter-donor variability when examining the human gut microbiota, it is possible that inoculating mice with feces from a different donor could generate a different response. However, pooling donors would produce artificial communities capable of unrealistic dietary responses.

Some of the observations from the animal study were also evident in the human intervention, with *Parabacteroides* abundance increased shortly after at the start of the intervention in the young, community, and long-stay groups, reaching statistically significant elevation in the latter two groups. *Clostridium* cluster IV abundance also increased soon after the start of the intervention in all three groups and was significantly elevated in the young controls and the long-stay elderly subjects. Several of the limited taxa identified as responsive in the human trial have previously been demonstrated to be affected by prebiotics. We observed increased abundance of the family *Ruminococcaceae*, well-characterized butyrate-producing bacteria, in long-stay subjects after prebiotic supplementation. *Ruminococcaceae* were positively associated with resistant starch intake in a pilot study of 14 obese males [2]. Similarly, *Phascolarctobacterium* was more abundant post-intervention in long-stay intervention subjects, and this genus was previously identified in healthy adult men consuming soluble corn fiber [3]. In contrast, the abundances of *Bifidobacterium*, *Lactobacillus*, *Dialister*, *Faecalibacterium*, and *Roseburia* were not altered by the supplementation, compared to the reported response of these bacteria to prebiotics [1, 3, 4, 7]. In the long-stay subjects, *Bifidobacterium* abundance actually decreased (Additional file 1: Figure S9). These findings can perhaps be explained by the quantity of ingredients in the prebiotic-supplemented regime with half of the prebiotic mix as resistant starch and the remaining half comprising the four other prebiotics. Furthermore, although we tested for dosage correlation, the differences in intervention dosage between long-stay subjects may have affected our ability to associate significant taxon changes with the intervention.

Our previous studies suggested that the gut microbiota may interact with the innate immune system leading to increased levels of inflammatory markers in long-stay subjects compared to community dwellers [15]. Supporting this hypothesis, we found an increased expression level of immune cell markers (CD11c, F4/80) and TNF-α levels, and a decrease of T cell CD8 expression in long-stay inoculated mice. These findings are not inconsistent with the fact that CD11c, F4/80, CD8, and TNF-α play an important role in host defense mechanisms against invading pathogens [32], and the relative number of CD8^+^ T cells decreases with age [33]. In the human trial, prebiotic supplementation in long-stay subjects led to decreased production of the chemokine CXCL11 that is produced in response to microbial antigens [34]. Modulation of the low diversity long-stay subject gut microbiome by prebiotic supplementation may thus impact the immune system, but the effects are clearly restricted in mediator range, and the health/clinical benefits are not clear.

We did not perform shotgun metagenomic sequencing of the gut microbiome of treated animals or human study participants, and so changes in the metabolic capacity of the microbiome or its host-interaction capability were not detectable. Strain level differences in the microbiome that we also could not detect can have important functional interactions with diet [35]. Some participants withdrew before finishing the study or were excluded due to low-quality sequencing reads. For valid clinical reasons, some subjects did not receive the same amount of prebiotic supplementation and we were not able to maintain the target dose of 21 g per day in long-stay subjects during the 6-month trial period. Compliance in the long-stay group was challenging particularly because of heightened sensitivity to bowel discomfort in less active subjects. Finally, the small number of subjects in the placebo groups especially in the long-stay cohort reduced statistical power, but this limitation was operationally imposed by the overall availability of eligible subjects especially because of the antibiotic treatment exclusion criterion in the older subjects.

## Conclusion

The high abundance taxa in community type gut microbiota were altered by a high-fat/low-fiber diet in mouse models and showed a progression toward a long-stay type gut microbiota structure. The long-stay type gut microbiota in the mouse model was perturbed by a high-fiber diet as evidenced by taxonomic alterations associated with the dietary change but did not move to a more community like structure. The combined evidence suggests an association between gut microbiota, diet, and immune response in animal models, but supplementation with prebiotics, at least with the supplementation levels we were able to get participant acceptance of in this study, had much weaker effects. It remains unclear whether ingredients in the prebiotic mix act alone or in combination to modulate the gut microbiome. Further studies are needed to optimize the choice of components combined in the prebiotic mix and to evaluate the effect of the prebiotic mix on the human gut microbiome in larger cohorts.

## Methods

### Human fecal inoculum preparation

Two human donors were selected from among community-dwelling and long-stay residential care groups based on their microbiota profiles from our previously published [15]. Fresh feces were collected from these donors for use in the murine intervention studies. Human stools were transferred to an anaerobic chamber within 1 min of voiding, transported to the lab in anaerobic bags, transferred to an anaerobic hood, and then diluted (ratio 1:10) in with pre-reduced PBS containing 20% glycerol, before being aliquoted in 1 ml volumes in cryovials and stored at − 80 °C. We compared these new samples to the donors’ previously analyzed samples to ensure the same typical community or long-stay microbiota profiles were observed (data not shown). The same community inocula and the same long-stay inocula were used for conventional mice and germ-free mice.

### Murine intervention study

The study was approved by the Animal Ethics Experimentation Committee of University College Cork (Approval number 2011/017). Two mouse models including conventional *C57* mice and germ-free Swiss Webster mice were used for three studies. A schematic overview of the experimental study design is presented in Fig. 1. First, conventional mice were treated with (1 g/l), vancomycin (500 mg/l), ciprofloxacin (200 mg/l), imipenem (250 mg/l), and metronidazole (1 g/l) as described previously [36] in the drinking water for 6 weeks and were fed ad libitum sterile standard chow which comprised 83.7% carbohydrate, 12.9% protein, and 2.5% fat (Special Diet Services, UK, code: 801010) (Additional file 3: Table S8) after 1 week of acclimation. In parallel, germ-free mice were fed standard chow diet till 3 weeks of age, then the customized diet until 6 weeks old. After a 24-h antibiotic-free period in conventional mice, all mice were inoculated with human microbiota derived from a healthy community subject or a frail long-stay subject (week 0) and maintained on a customized diet corresponding to community or long-stay residence (community/long-stay-based diet) with ad libitum access to food (8 g/day) for 4 weeks. Animal weight and food intake were recorded. Then mice were assigned to the opposite diet for a further four weeks, until terminal sacrifice at week 9, in a crossover design. The third study was conducted with conventional mice, but they were sacrificed at week 4 for a non-crossover design. Fecal samples were collected from each animal at several time points: before the antibiotic treatment (week − 7) for conventional mice, end of week 1, end of week 4, end of week 5, end of week 8. For some animals, and a minority of timepoints, fecal samples could not be collected. After sacrifice, luminal content from the caecum and colon, and colonic scrapings, were collected for microbiome analysis and spleen was isolated immediately for analysis of the immune response. Spleen cells were stimulated for tumor necrosis factor alpha (TNF-α) analysis and processed for flow cytometric analysis as described in Additional file 4: Supplementary Methods.

Community/long-stay-based diets (as described in Table 1) were formulated to mimic a low-fat/high-fiber diet typical of healthy community-dwelling older people and a high-fat/low-fiber diet of long-term residential care older people from our previous findings [15], albeit with artificially high-fiber content. Median frequencies of consumption were calculated for the selected diet from each group and the UK institutionalized and aged 65+ portion sizes applied [37]. Dietary analysis was conducted using WISP© (Weighed Intake Software Program; Tinuviel Software, Warrington, UK).

### Human study subject recruitment and sample collection

Ethical approval and exclusion criteria used for subject selection are described in Additional file 4: Supplementary Methods. Subjects in each cohort were randomized to receive either a dietary supplement as an intervention product, based on ingredients procured by General Mills Inc., or a placebo (maltodextrin) manufactured by Nutrition Supplies Services, Cork, Ireland. The intervention dietary supplement provided to participants with a prebiotic mix (21 g) including wheat dextrin (3 g), resistant starch (10 g), polydextrose (3 g), soluble corn fiber (3 g), and galactooligo-saccharide (2 g), as per Table 2. A dietary prebiotic supplement was administered to the participants. All subjects were instructed (or aided) to take the supplement dose in the morning with food over 26 weeks. Each dose consisted of 10.5 g of the placebo/prebiotic powder. Subjects in the long-stay cohort who were reported having a lower baseline dietary fiber intake [38] took an additional dose of 10.5 g powder, equating to a daily dose of 21 g powder.

Samples obtained from participants included stool, whole blood, serum, urine, anthropometric measurements, and clinical history at four time points: baseline (week 0), halfway (week 13), end of study (week 26), and 6 weeks after the cessation of treatment (week 32). A food frequency questionnaire (FFQ) was utilized to calculate HFD index [18]. Non-fasted blood samples were analyzed at Cork University Hospital clinical laboratories. In addition, whole and coagulated bloods were collected from all of the participants to further determine inflammation and immune response, for the following tests: surface staining for CD4 and CD8, serum for CRP, lipopolysaccharide (LPS) assay, cytokines (IL-17, IFN-γ, IL-10, IL-4, IL-6), and chemokines (CXCL11, CCL11). See Additional file 4: Supplementary Methods for details**.**

### 16S rRNA gene sequencing, bioinformatic, and statistical analysis

Microbial DNA was extracted, amplified, and sequenced from fecal samples of mice and human as previously [39] and further described in Additional file 4: Supplementary Methods. All statistical analyses were performed using R [40], described in Additional file 4: Supplementary Methods, and briefly here. Differences in beta diversity were investigated using analysis of similarity (ANOSIM). Changes in alpha diversity were detected using linear mixed models (R package *nlme* [41]). Analysis of variance (ANOVA) tests were used to investigate dosage difference effects on alpha diversity in linear mixed models. Differences in alpha diversity among cohorts were compared by Wilcoxon signed rank test (paired data), the Mann-Whitney *U* test (unpaired data), or the Kruskal-Wallis test with Dunn’s multiple comparison test. Differential abundance at phylum and genus level among time points within treatment groups was identified using DESeq2 [42] controlling for subject identity across time-points and HFD-index differences, and adjusted using Benjamini–Hochberg correction.

## Additional files

Additional file 1: Supplementary **Figures S1–S11.** (PDF 12337 kb)

Additional file 2: Supplementary **Tables S1–S4.** (DOCX 30 kb)

Additional file 3: Supplementary **Tables S5–S8.** (XLSX 51 kb)

Additional file 4: Supplementary Methods. (DOCX 38 kb)

## Abbreviations

ANOSIM: Analysis of similarity
ANOVA: Analysis of variance
CAGs: Co-abundance groups
CM: Community
CRP: C-reactive protein
FFQ: Food frequency questionnaire
HFD: Healthy food diversity
LPS: Lipopolysaccharide
LS: Long-stay
LTA: Lipoteichoic acid
PCoA: Principal coordinates analysis
SCFAs: Short-chain fatty acids
T0: Baseline (week 0)
T13: Halfway (week 13)
T26: End of study (week 26)
T32: Six weeks after the cessation of treatment (week 32)
YH: Young-healthy
