# Supplementary material for: Prebiotic supplementation in frail older people affects specific gut microbiota taxa but not global diversity
Source: Microbiome. 2019 Mar 13;7:39. doi: 10.1186/s40168-019-0654-1 (PMC6417215; doi:10.1186/s40168-019-0654-1)
Supplement: Supplementary file 1 — Supplementary Figures S1–S11. (PDF 12337 kb) [file 40168_2019_654_MOESM1_ESM.pdf]

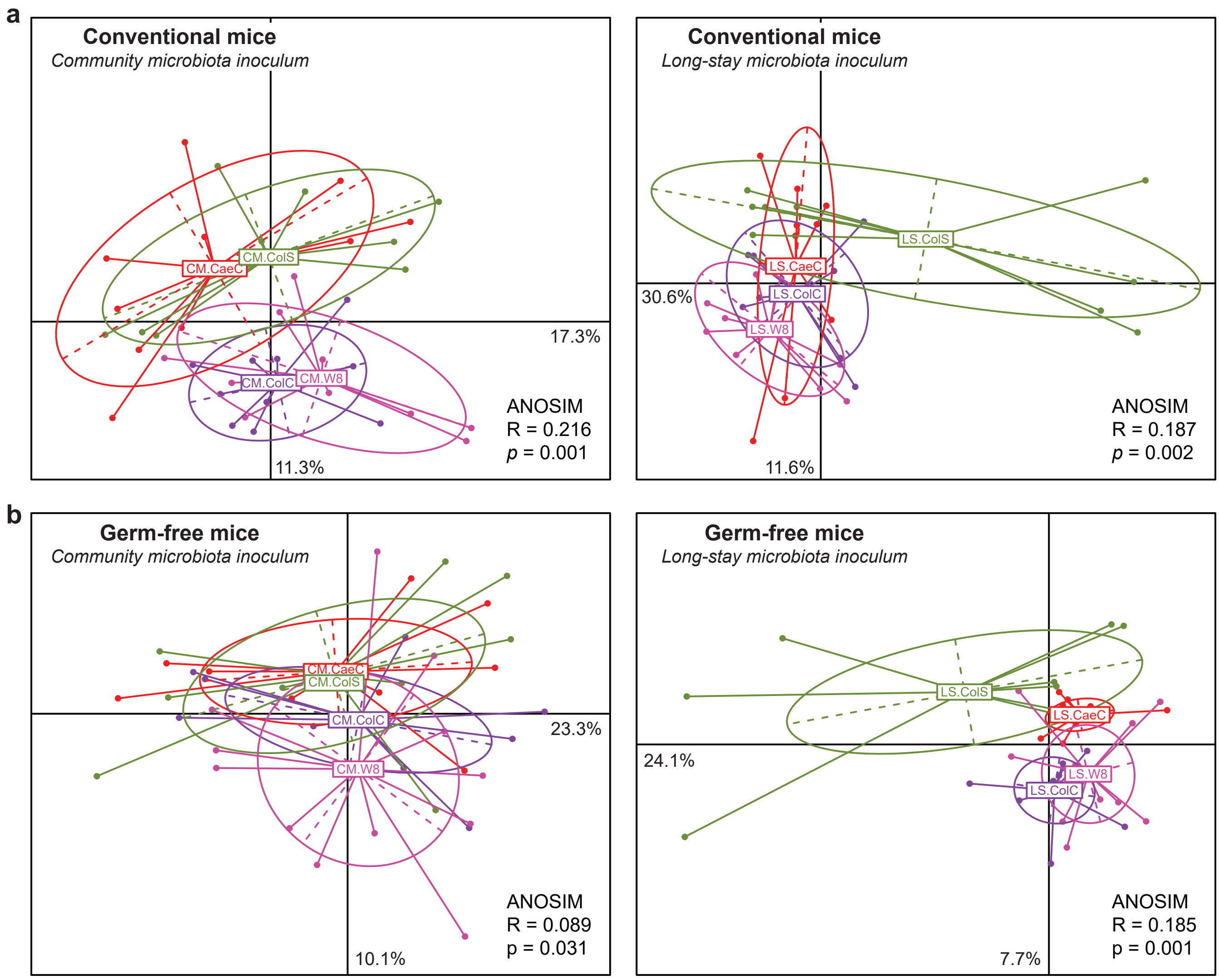

Figure S1. Principle coordinates analysis plots comparing bacterial community structure between faeces, colonic scrapings, colonic contents and caecal contents at week 8 based on Spearman distances. (**a**) Samples from conventional mice cross over design. (**b**) Samples from germ-free mice cross over design. The significant differences between groups were calculated by analysis of similarity (ANOSIM) tests. Abbreviations: CM, Community; LS, Long-stay; W8, week 8 (feaces); ColS, colonic scraping; ColC, colonic content; CaeC, caecal content.

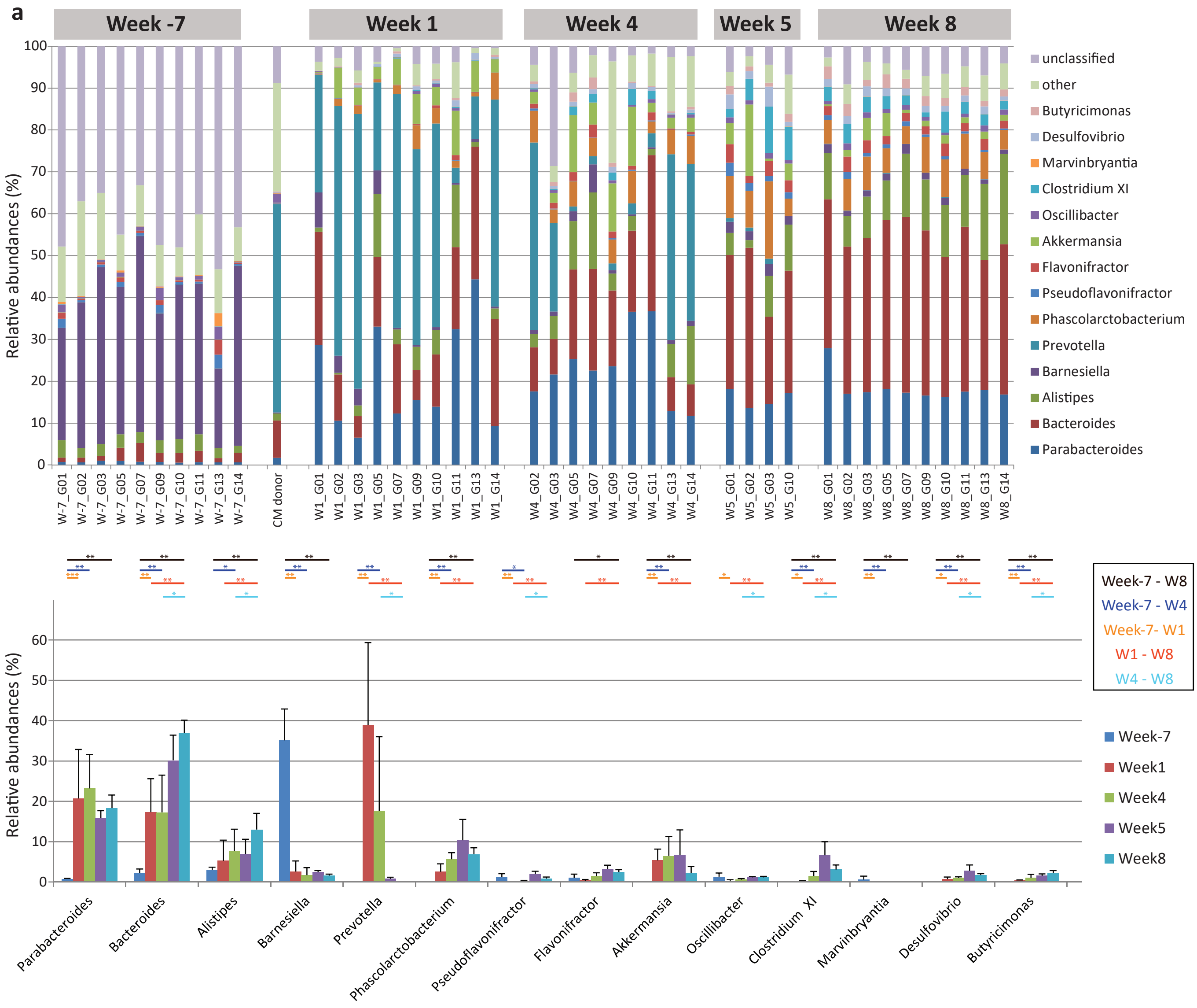
a
Week -7
Week 1
Week 4
Week 5
Week 8
Relative abundances (%)
unclassified
other
Butyricimonas
Desulfovibrio
Marvinbryantia
Clostridium XI
Oscillibacter
Akkermansia
Flavonifractor
Pseudoflavonifractor
Phascolarctobacterium
Prevotella
Barnesiella
Alistipes
Bacteroides
Parabacteroides
CM donor
Week-7 - W8
Week-7 - W4
Week-7- W1
W1 - W8
W4 - W8
Week-7
Week1
Week4
Week5
Week8
Parabacteroides
Bacteroides
Alistipes
Barnesiella
Prevotella
Phascolarctobacterium
Pseudoflavonifractor
Flavonifractor
Akkermansia
Oscillibacter
Clostridium XI
Marvinbryantia
Desulfovibrio
Butyricimonas

Figure S2a.

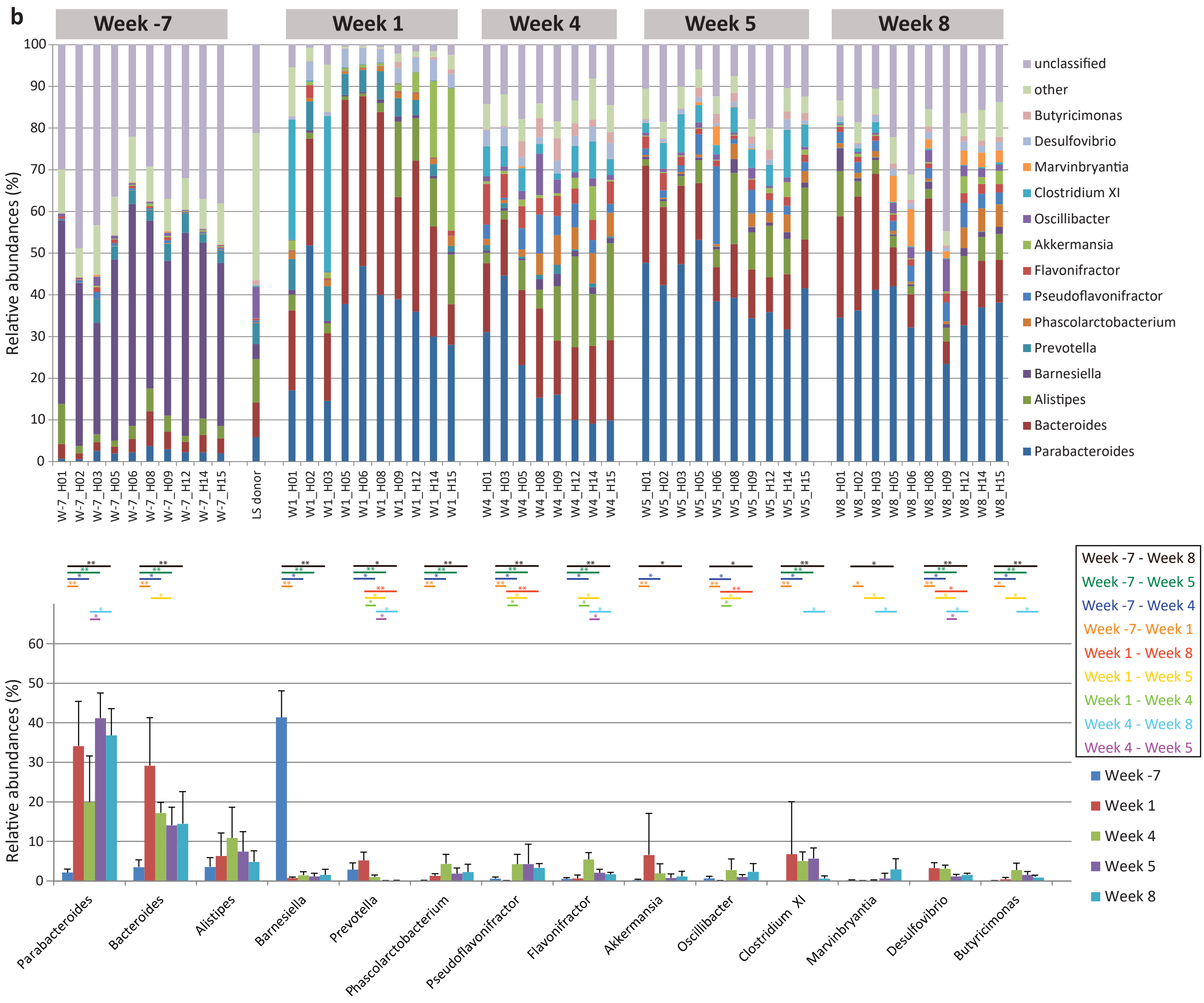

Figure S2. Gut microbiota composition at the genus level of conventional mice inoculated with a community microbiota (**a**) or with a long-stay microbiota (**b**), grouped by time points. *P*-values were calculated using Wilcoxon signed-rank tests and were corrected for the multiple comparison using the Benjamini–Hochberg adjustment, *p < 0.05, **p < 0.01, ***p < 0.001. Abbreviations: CM donor, healthy community subject; LS donor, frail long-stay subject; and Week -7 (week minus 7), murine microbiota before antibiotic treatment.

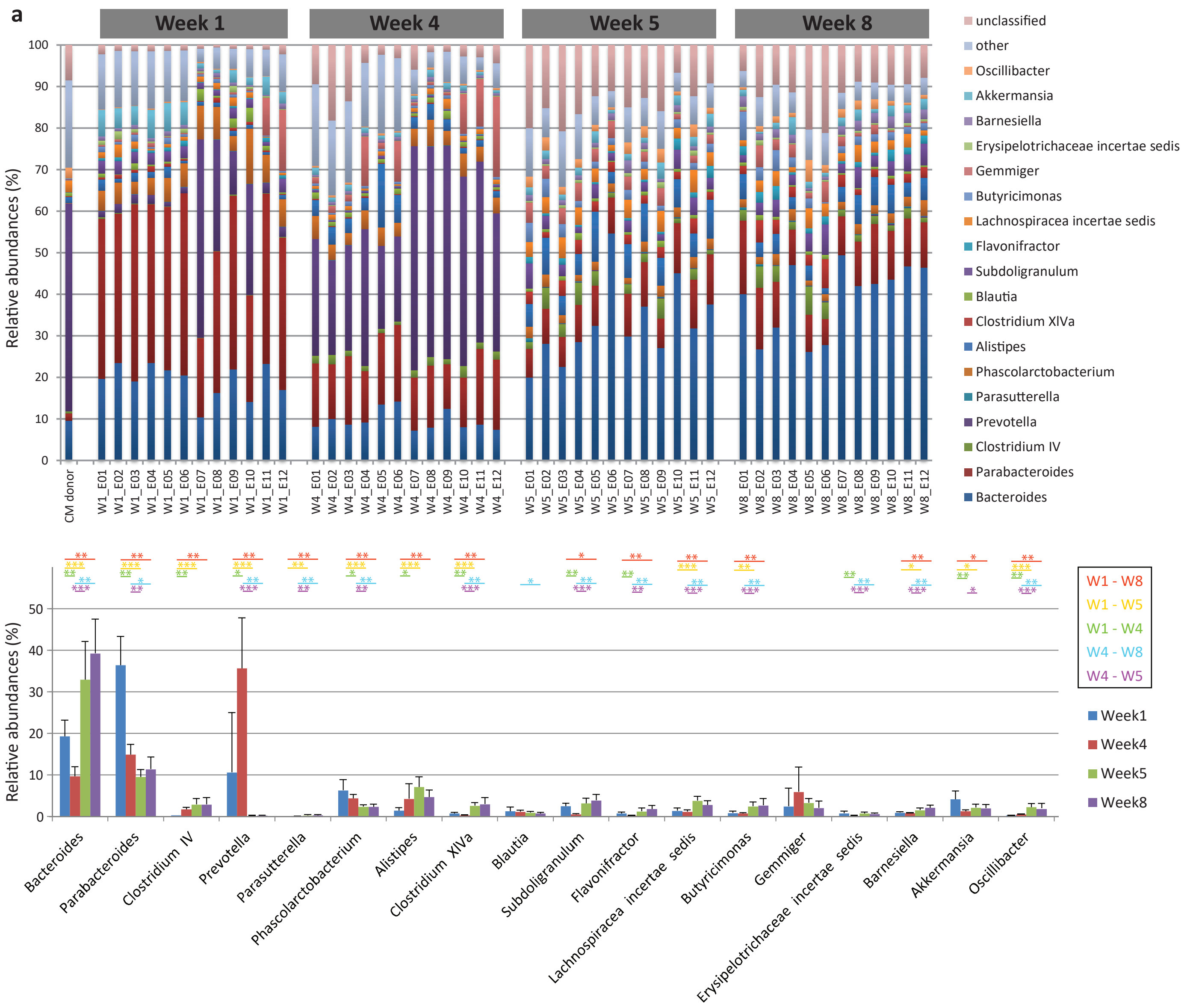
a
Week 1
Week 4
Week 5
Week 8
Relative abundances (%)
CM donor
unclassified
other
Oscillibacter
Akkermansia
Barnesiella
Erysipelotrichaceae incertae sedis
Gemmiger
Butyricimonas
Lachnospiracea incertae sedis
Flavonifractor
Subdoligranulum
Blautia
Clostridium XlVa
Alistipes
Phascolarctobacterium
Parasutterella
Prevotella
Clostridium IV
Parabacteroides
Bacteroides
W1 - W8
W1 - W5
W1 - W4
W4 - W8
W4 - W5
Week1
Week4
Week5
Week8
Relative abundances (%)
Bacteroides
Parabacteroides
Clostridium IV
Prevotella
Parasutterella
Phascolarctobacterium
Alistipes
Clostridium XlVa
Blautia
Subdoligranulum
Flavonifractor
Lachnospiracea incertae sedis
Butyricimonas
Gemmiger
Erysipelotrichaceae incertae sedis
Barnesiella
Akkermansia
Oscillibacter

Figure S3a.

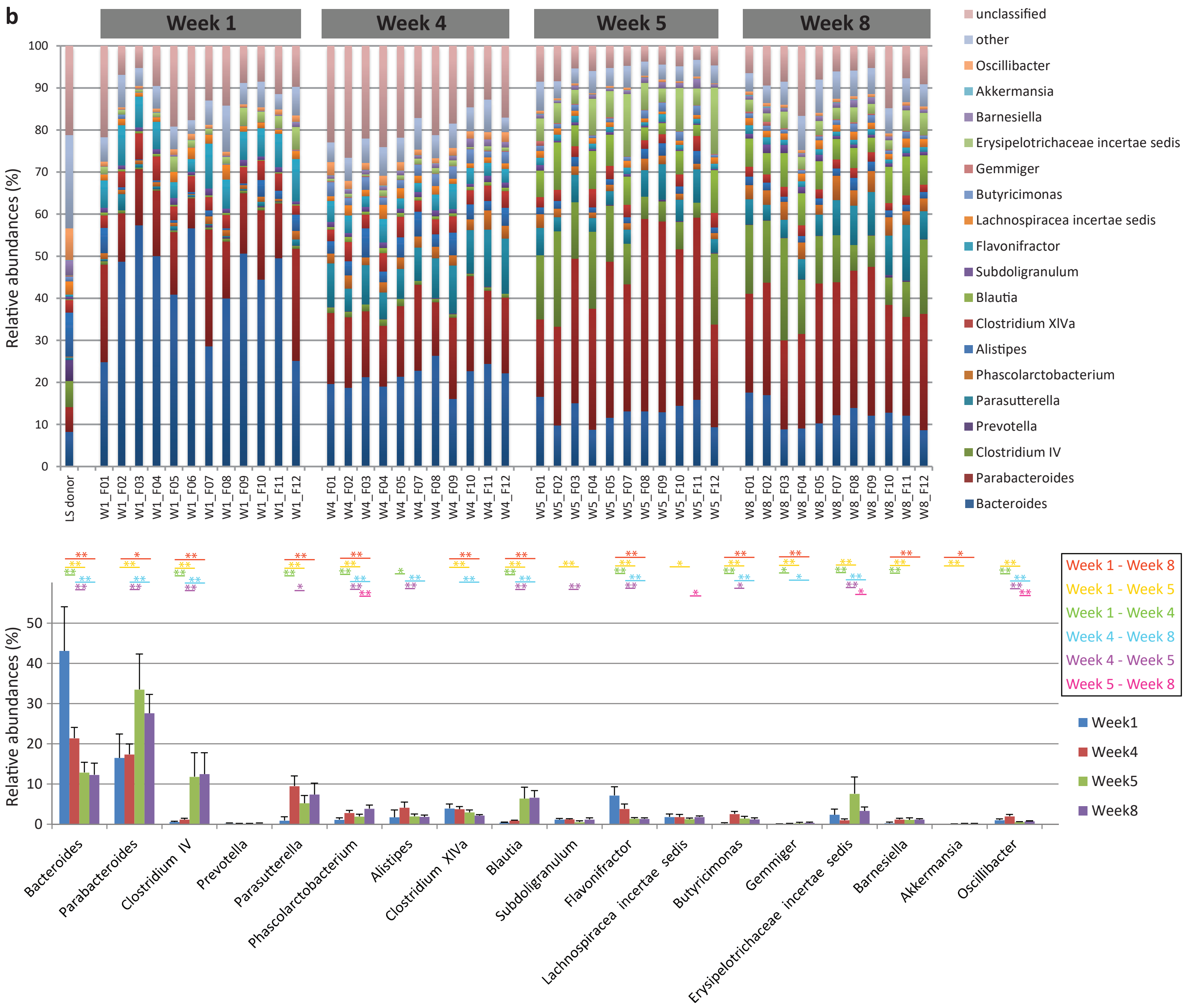

Figure S3. Gut microbiota composition at the genus level of germ-free mice inoculated with a community microbiota (**a**) or with a long-stay microbiota (**b**), grouped by time points. *P*-values were calculated using Wilcoxon signed-rank tests and were corrected for the multiple comparison using the Benjamini–Hochberg adjustment, $*p < 0.05$, $**p < 0.01$, $***p < 0.001$. Abbreviations: CM donor, healthy community subject; LS donor, frail long-stay subject.

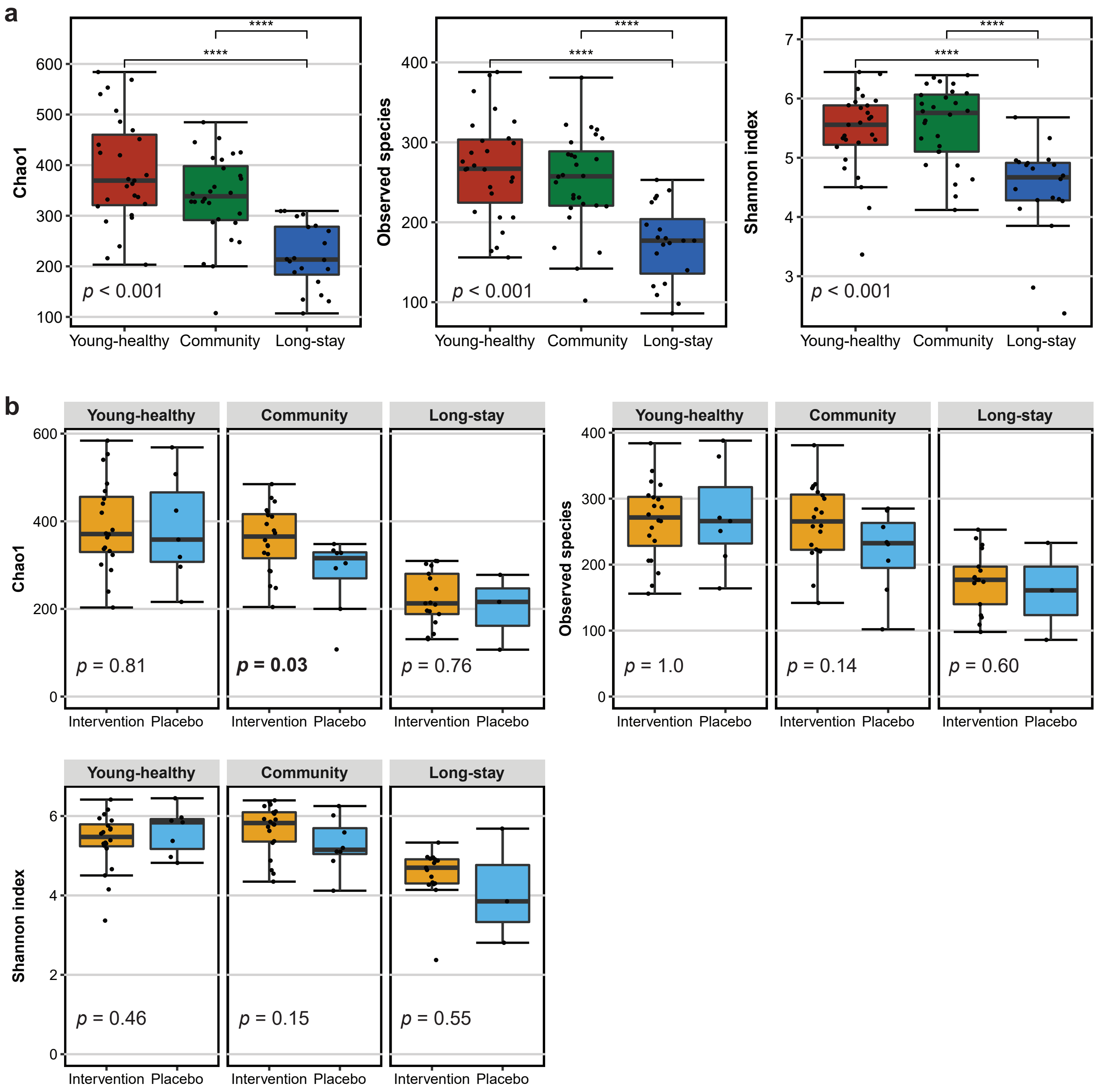

Figure S4. Alpha diversity comparisons of the human gut microbiome at baseline (T0), grouped by residence location (**a**) or residence location and treatment group (**b**). *P*-values were calculated using Kruskal-Wallis test for for three independent groups and Mann-Whitney U tests for unpaired groups, ***$p < 0.001$.

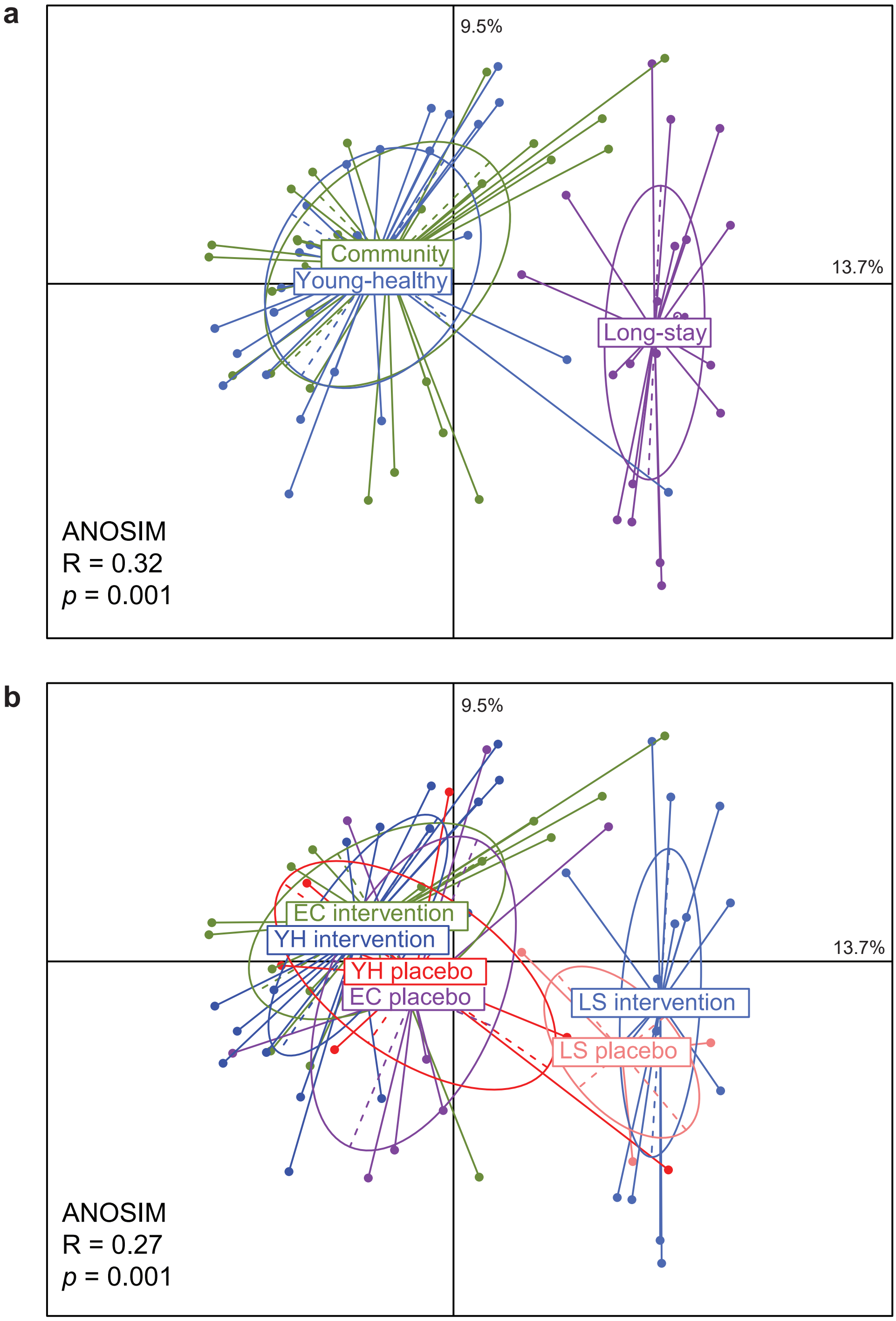

Figure S5. Beta diversity comparisons based on Bray-Curtis distances of the human gut microbiome at baseline (T0), grouped by residence location (**a**) or residence location and treatment group (**b**). The significant differences between groups were calculated by ANOSIM tests. Abbreviations: Abbreviations: YH, Young-healthy; CM, Community; LS, Long-stay.

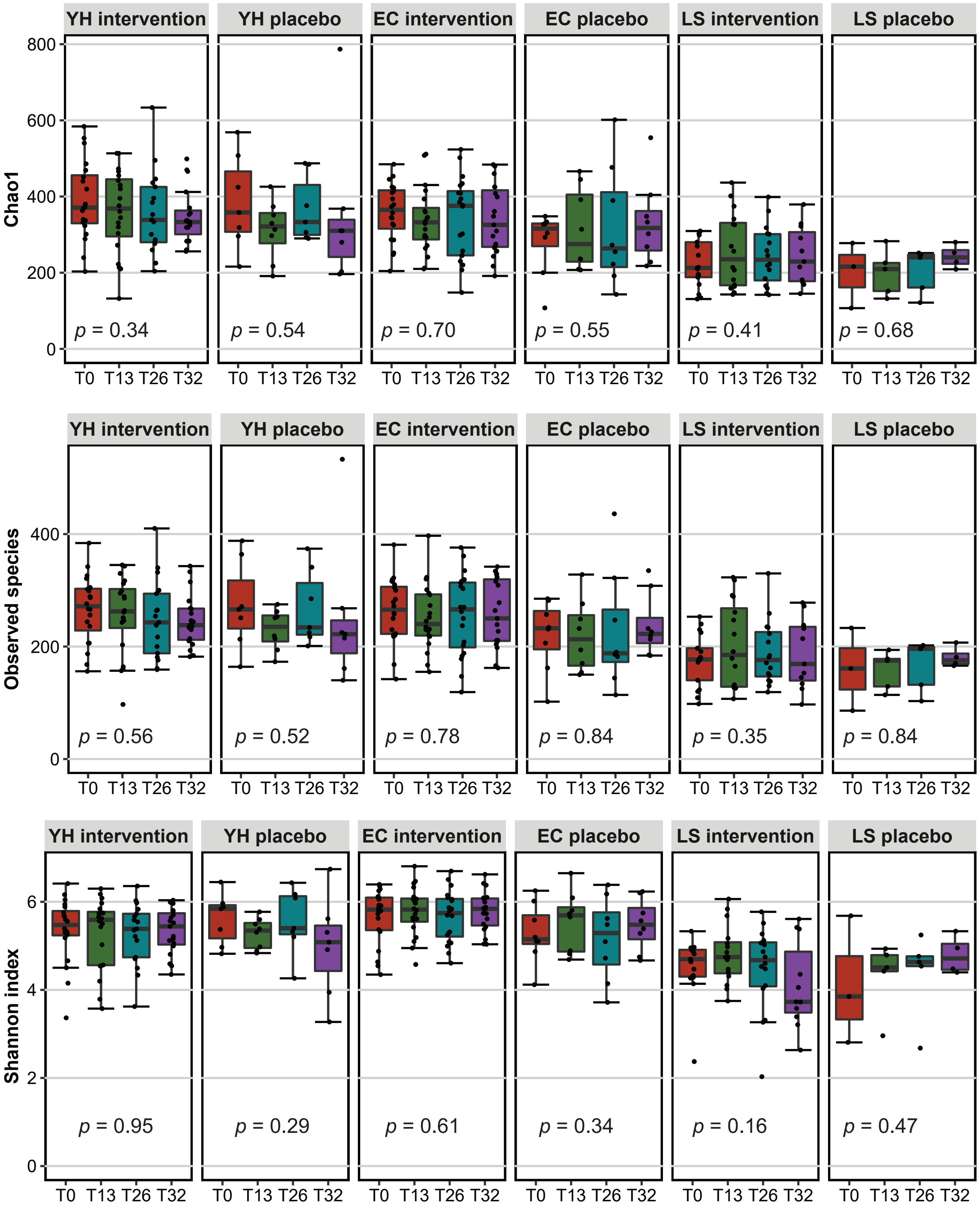

Figure S6. Boxplot of bacterial alpha-diversity indices for the 16S rRNA gene sequences between different time points in each treatment groups. *P*-values were calculated from linear mixed effects models. Abbreviations: YH, Young-healthy; CM, Community; LS, Long-stay; T0, baseline (week 0); T13, halfway (week 13); T26, end of study (week 26); and T32, six weeks after the cessation of treatment (week 32).

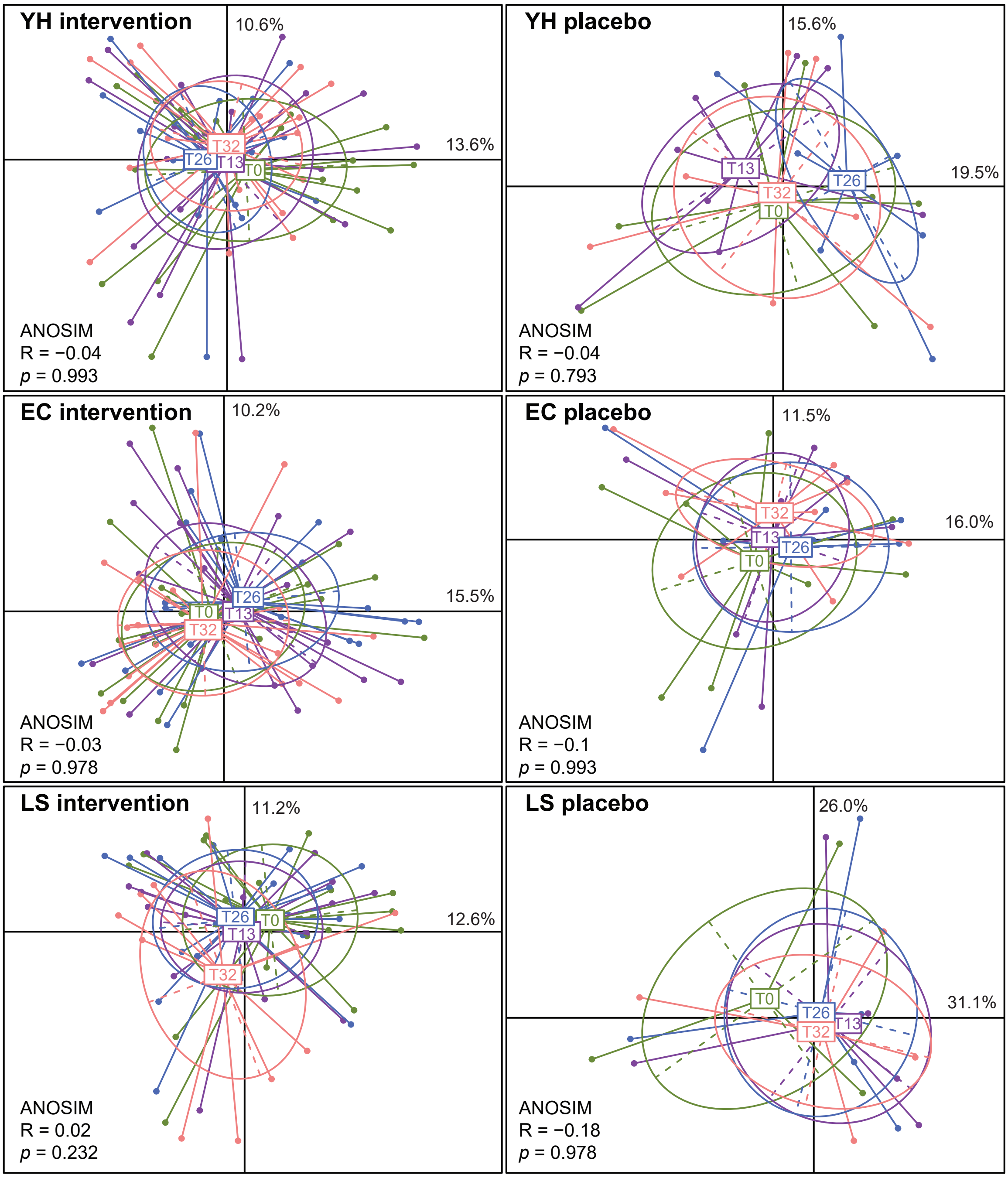

Figure S7. Principle Coordinates Analysis based on Bray-Curtis distances of partial sequences of bacterial 16S rRNA genes from human faecal samples of treatment groups, grouped by time points. The significant differences between groups were calculated by ANOSIM tests. Abbreviations: YH, Young-healthy; CM, Community; LS, Long-stay; T0, baseline (week 0); T13, halfway (week 13); T26, end of study (week 26); and T32, six weeks after the cessation of treatment (week 32).

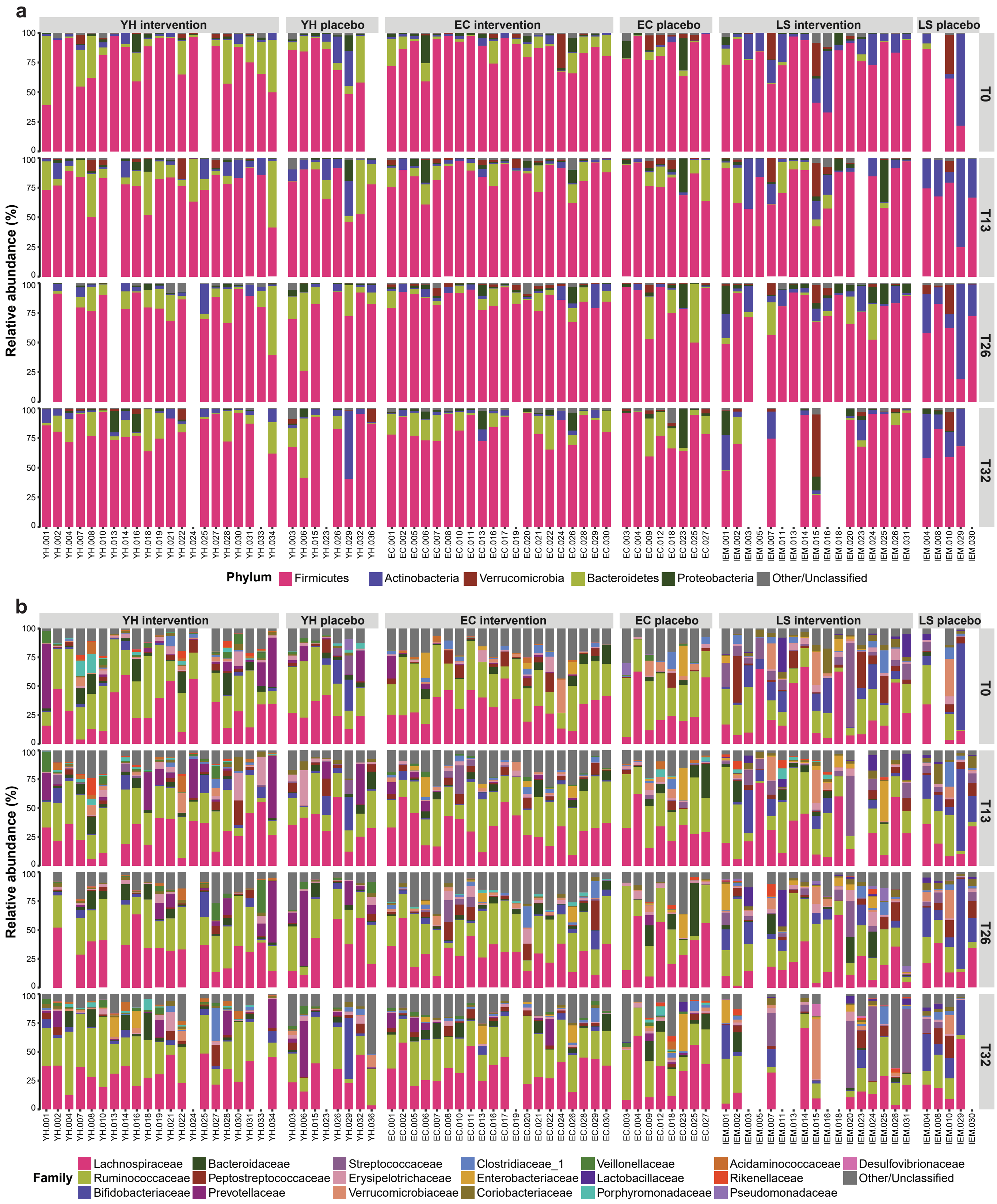

Figure S8. Relative abundance of faecal microbiota taxa at (**a**) phylum level, (**b**) family level in subjects, grouped according to treatment groups and time points. Abbreviations: YH, Young-healthy; CM, Community; LS, Long-stay; T0, baseline (week 0); T13, halfway (week 13); T26, end of study (week 26); and T32, six weeks after the cessation of treatment (week 32).

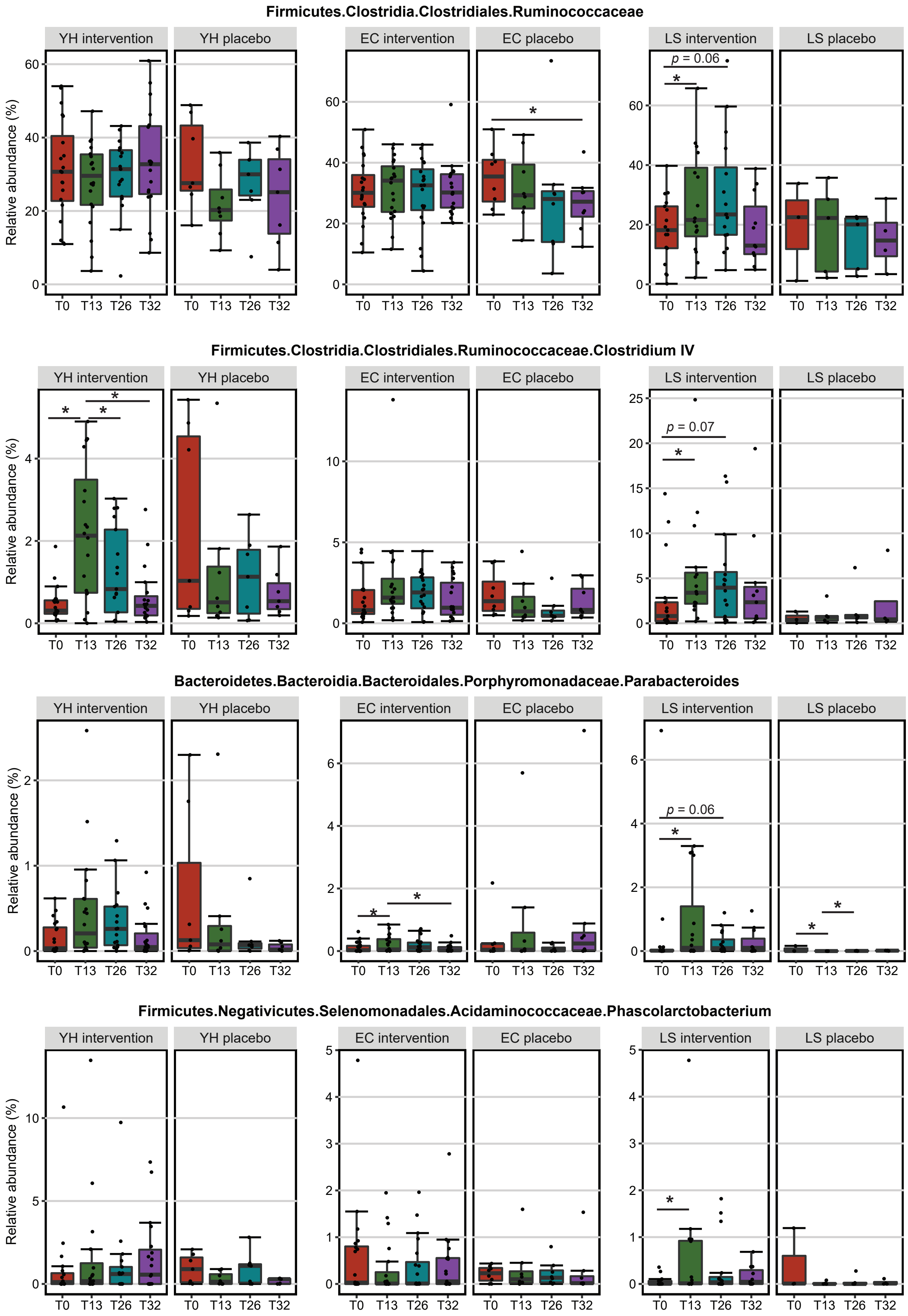

Firmicutes.Clostridia.Clostridiales.Ruminococcaceae
Firmicutes.Clostridia.Clostridiales.Ruminococcaceae.Clostridium IV
Bacteroidetes.Bacteroidia.Bacteroidales.Porphyromonadaceae.Parabacteroides
Firmicutes.Negativicutes.Selenomonadales.Acidaminococcaceae.Phascolarctobacterium
YH intervention
YH placebo
EC intervention
EC placebo
LS intervention
LS placebo
Relative abundance (%)
T0
T13
T26
T32
p = 0.06
p = 0.07
*

Figure S9.

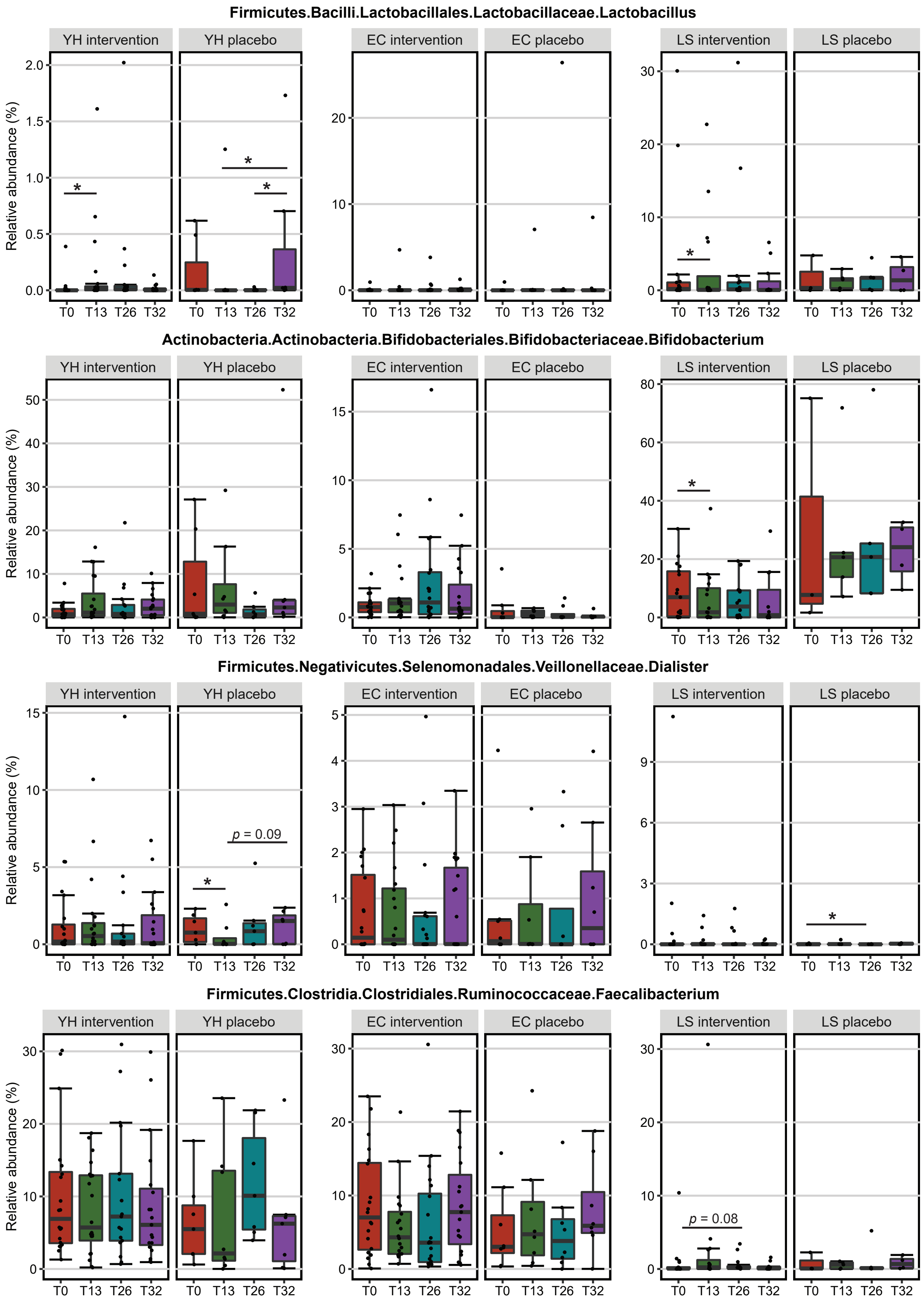

Supplementar **Figure S 9.** Box plot of relative abundances of the statistically significantly different taxa after treatment. Adjusted *p*-values were calculated by DESeq2 tests with Benjamini–Hochberg correction, *$p < 0.05$. Abbreviations: YH, Young-healthy; CM, Community; LS, Long-stay; T0, baseline (week 0); T13, halfway (week 13); T26, end of study (week 26); and T32, six weeks after the cessation of treatment (week 32).

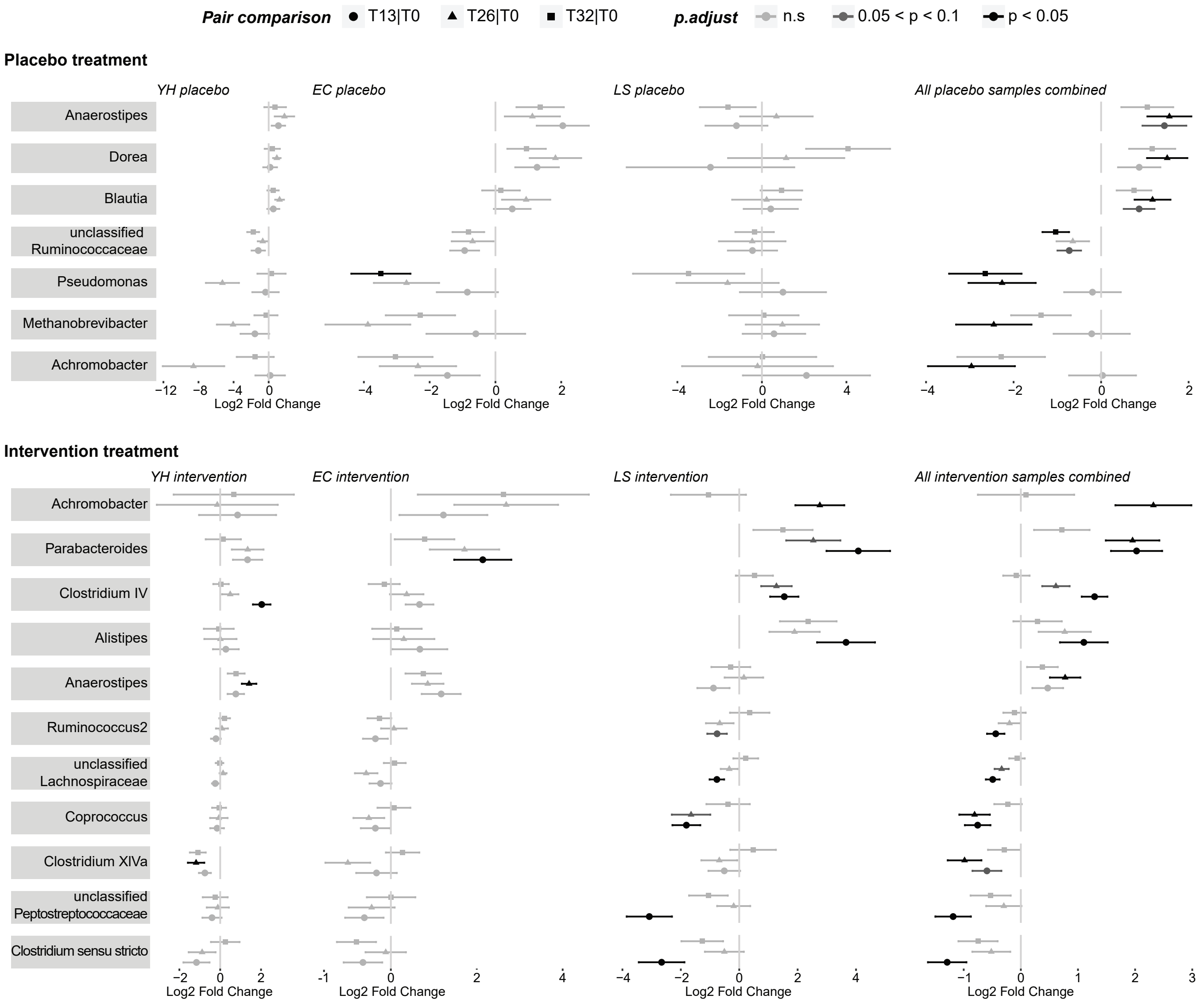

Figure S10. Genera that were significantly increased (log2 fold change > 0) or decrease (log2 fold change < 0) in the placebo/prebiotic intervention to baseline at T0 across all available intervention or placebo samples, compared with the results from the individual cohorts. Adjusted *p*-values were calculated by DESeq2 test with Benjamini–Hochberg correction. Abbreviations: YH, Young-healthy; CM, Community; LS, Long-stay. T0, baseline (week 0); T13, halfway (week 13); T26, end of study (week 26); and T32, six weeks after the cessation of treatment (week 32).

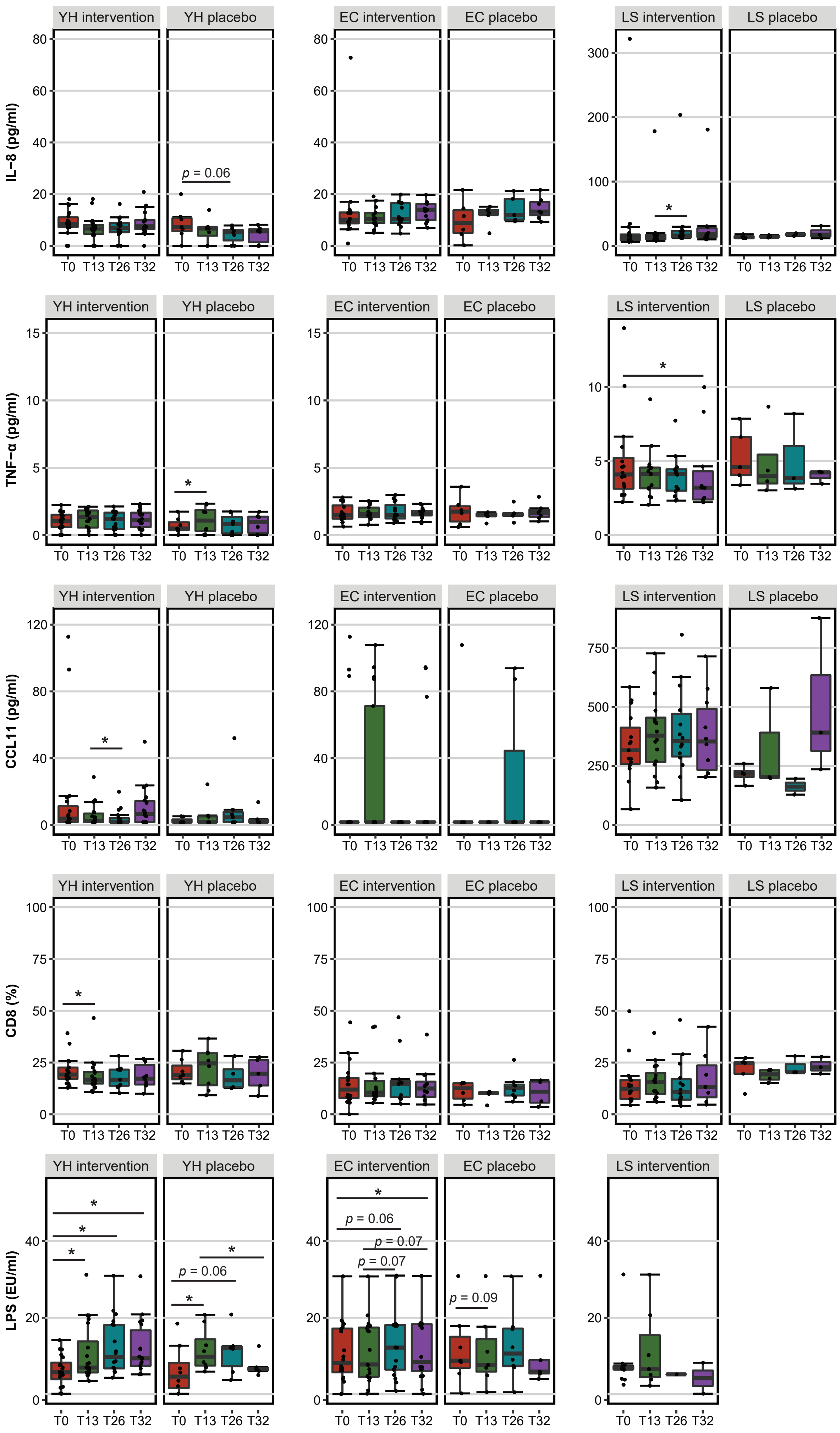

YH intervention
YH placebo
EC intervention
EC placebo
LS intervention
LS placebo
IL−8 (pg/ml)
TNF−α (pg/ml)
CCL11 (pg/ml)
CD8 (%)
LPS (EU/ml)
T0 T13 T26 T32
p = 0.06
*
p = 0.07
p = 0.09

Figure S11.

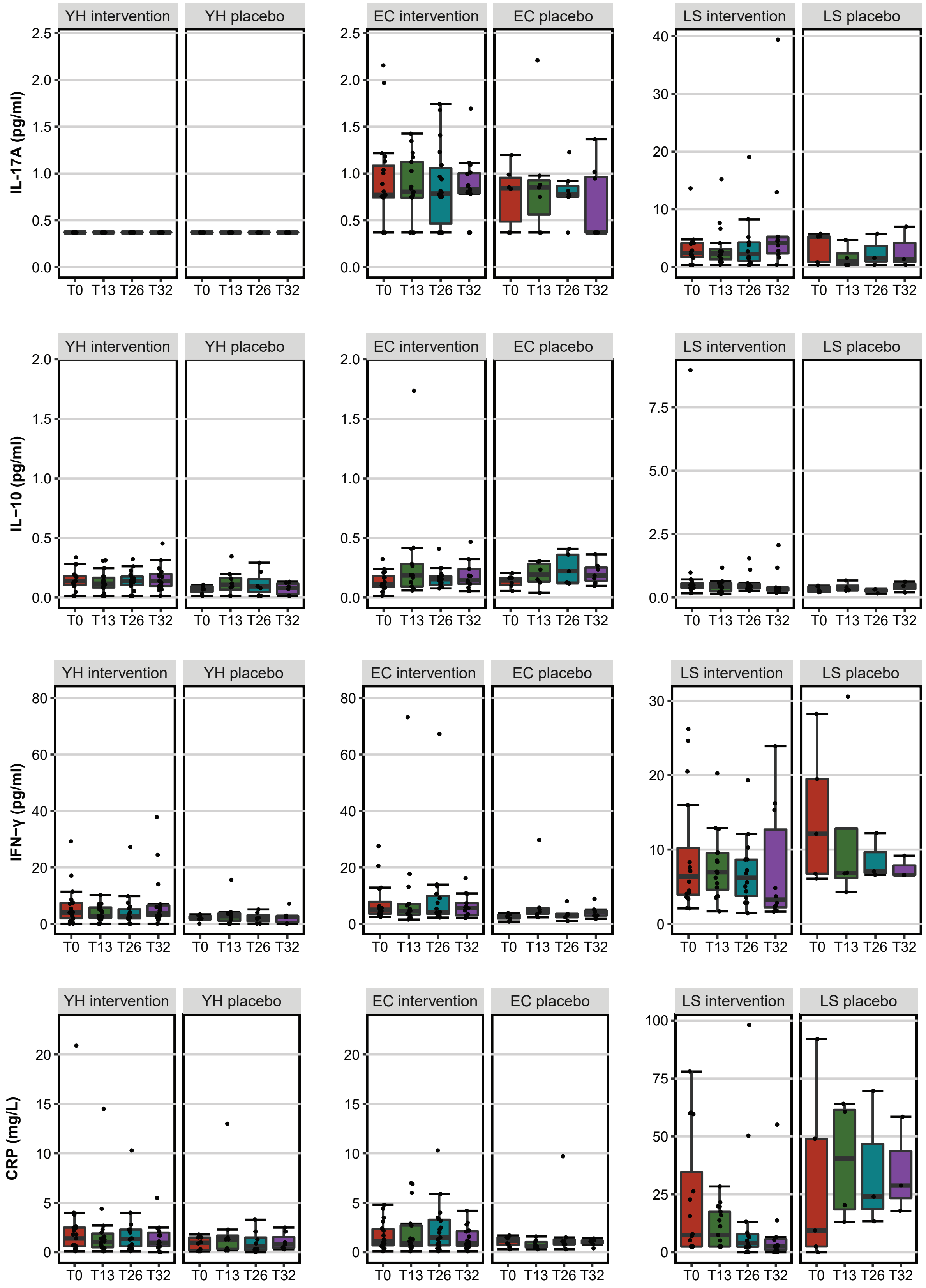

Figure S11. Immunological/inflammatory profiling between time points within treatment groups. *P*-values were calculated from Wilcoxon signed-rank tests, $*p < 0.05$. Abbreviations: YH, Young-healthy; CM, Community; LS, Long-stay; T0, baseline (week 0); T13, halfway (week 13); T26, end of study (week 26); and T32, six weeks after the cessation of treatment (week 32).
