# Supplementary material for: Prebiotic supplementation in frail older people affects specific gut microbiota taxa but not global diversity
Source: Microbiome. 2019 Mar 13;7:39. doi: 10.1186/s40168-019-0654-1 (PMC6417215; doi:10.1186/s40168-019-0654-1)
Supplement: Supplementary file 2 — Supplementary Tables S1–S4. (DOCX 30 kb) [file 40168_2019_654_MOESM2_ESM.docx]

**Table S1.** Number of the human gut microbial genera transferred into conventional mice and germ-free mice

|  | **Long-stay donor** | | **Community donor** | |
| --- | --- | --- | --- | --- |
|  | Conventional | Germ-free | Conventional | Germ-free |
| ***Genera found*** |  |  |  |  |
| In inoculum and any mouse | 45 | 46 | 37 | 33 |
| *In inoculum and at least 50% of mice* | *26* | *36* | *22* | *27* |
| In inoculum but not in any mice | 9 | 8 | 9 | 13 |
| Not in inoculum but in any mice | 26 | 6 | 21 | 18 |
| *Not in inoculum but in 50% of mice* | *1* | *4* | *4* | *10* |

**Table S2.** Subjects withdrawn from clinical trial

|  |  | **Young-healthy** | | **Community** | | **Long-stay** | | **Total** |
| --- | --- | --- | --- | --- | --- | --- | --- | --- |
|  |  | Intervention | Placebo | Intervention | Placebo | Intervention | Placebo |  |
| ***Number of subjects at each time points*** | | | | | | | |  |
|  | T0 | 23 | 10 | 21 | 8 | 23 | 8 | 93 |
|  | T13 | 20 | 8 | 20 | 8 | 20 | 6 | 82 |
|  | T26 | 19 | 7 | 20 | 8 | 17 | 5 | 76 |
|  | T32 | 18 | 7 | 20 | 8 | 11 | 5 | 69 |
| ***Reason for withdrawal*** | | | | | | | |  |
|  | Withdraw at the investigators discretion | 1 | 0 | 0 | 0 | 3 | 1 | 5 |
|  | Unspecified personal reasons | 4 | 1 | 1 | 0 | 3 | 0 | 9 |
|  | Loss to follow up | 0 | 2 | 0 | 0 | 0 | 0 | 2 |
|  | Unable to produce feacal samples | 0 | 0 | 0 | 0 | 2 | 1 | 3 |
|  | Died of unrelated causes | 0 | 0 | 0 | 0 | 4 | 1 | 5 |

**Table S3.** Counts of participants per treatment group and time point for whom a minimum of 7,284 high-quality sequence reads could be obtained.

| **Residence location** | | **Time point** | | | |
| --- | --- | --- | --- | --- | --- |
|  |  | **T0** | **T13** | **T26** | **T32** |
| Young Healthy (YH)  (n = 29) | YH intervention | 20 | 20 | 17 | 19 |
|  | YH placebo | 7 | 8 | 7 | 7 |
| Community (EC)  (n = 28) | EC intervention | 20 | 20 | 20 | 19 |
|  | EC placebo | 8 | 8 | 8 | 8 |
| Long Stay (LS)  (n=22) | LS intervention | 17 | 16 | 16 | 11 |
|  | LS placebo | 3 | 5 | 5 | 4 |

**Table S4.** ANOVA comparing two linear mixed models, alpha diversity ~ time points and alpha diversity ~ time points + dosage, in each intervention treatment groups with subjects as random intercept.

| **Treatment group** | **Variable** | **Model** | **Group effects** | **AIC** | **BIC** | **logLik** | **Test** | **L.Ratio** | ***p* value** |
| --- | --- | --- | --- | --- | --- | --- | --- | --- | --- |
| YH intervention | Chao1 | 1 | Timepoints | 915.0 | 929.0 | -451.5 |  |  |  |
|  |  | 2 | Timepoints + Dosage | 916.6 | 933.0 | -451.3 | 1 vs 2 | 0.364 | 0.546 |
|  | Observed species | 1 | Timepoints | 843.1 | 857.0 | -415.5 |  |  |  |
|  |  | 2 | Timepoints + Dosage | 844.9 | 861.2 | -415.4 | 1 vs 2 | 0.174 | 0.677 |
|  | Shannon index | 1 | Timepoints | 163.5 | 177.5 | -75.7 |  |  |  |
|  |  | 2 | Timepoints + Dosage | 165.0 | 181.3 | -75.5 | 1 vs 2 | 0.532 | 0.466 |
| EC intervention | Chao1 | 1 | Timepoints | 920.0 | 934.2 | -454.0 |  |  |  |
|  |  | 2 | Timepoints + Dosage | 921.4 | 938.0 | -453.7 | 1 vs 2 | 0.599 | 0.439 |
|  | Observed species | 1 | Timepoints | 845.3 | 859.5 | -416.7 |  |  |  |
|  |  | 2 | Timepoints + Dosage | 845.4 | 862.0 | -415.7 | 1 vs 2 | 1.866 | 0.172 |
|  | Shannon index | 1 | Timepoints | 116.0 | 130.2 | -52.0 |  |  |  |
|  |  | 2 | Timepoints + Dosage | 117.6 | 134.1 | -51.8 | 1 vs 2 | 0.432 | 0.511 |
| LS intervention | Chao1 | 1 | Timepoints | 695.0 | 707.6 | -341.5 |  |  |  |
|  |  | 2 | Timepoints + Dosage | 693.6 | 708.2 | -339.8 | 1 vs 2 | 3.433 | 0.064 |
|  | Observed species | 1 | Timepoints | 660.9 | 673.5 | -324.5 |  |  |  |
|  |  | 2 | Timepoints + Dosage | 660.9 | 675.5 | -323.4 | 1 vs 2 | 2.085 | 0.149 |
|  | Shannon index | 1 | Timepoints | 151.6 | 164.2 | -69.8 |  |  |  |
|  |  | 2 | Timepoints + Dosage | 149.9 | 164.6 | -68.0 | 1 vs 2 | 3.671 | 0.055 |

*Note. AIC = Akaike's information criterion corrected for sample size; BIC: Baysesian information critetian; logLik: log restricted maximum likehood, L.Ratio: likelihood ratio test comparing two models.*
