# Supplementary material for: Prebiotic supplementation in frail older people affects specific gut microbiota taxa but not global diversity
Source: Microbiome. 2019 Mar 13;7:39. doi: 10.1186/s40168-019-0654-1 (PMC6417215; doi:10.1186/s40168-019-0654-1)
Supplement: Supplementary file 4 — Supplementary Methods. (DOCX 38 kb) [file 40168_2019_654_MOESM4_ESM.docx]

# Supplementary Methods

**Mouse splenocyte stimulation for TNF-α production**

Harvested mouse splenocytes were prepared according to the protocol by Davies *et. al*. [1]. The splenocytes of each animal were seeded, in duplicate, into 96-well round bottomed tissue culture plates at a concentration of 1 × 10^5^ cells per well in a volume of 0.2 ml. They were stimulated for tumour necrosis factor alpha (TNF-α) with RPMI-1640 (Sigma Aldrich Ireland) complete medium (control), or with bacterial surface components including lipopolysaccharide from *Escherichia coli* K12 (LPS-K12 100 ng/ml) (Invivogen), lipoteichoic acid from *Staphylococcus aureus* (LTA-SA 500 ng/ml) (InvivoGen), or LTA from *Bacillus subtilis* (LTA-BS 500 ng/ml) (InvivoGen). The complete medium consisted of 13% RPMI medium, 10% faecal bovine serum, 1% penicillin/streptomycin, 1% vitamins and 1% non-essential amino acid solution. All reagents were from Sigma Aldrich, Ireland. Plates were incubated at 37°C and 5% CO_2_ for 24 h, and supernatants stored at -80°C for subsequent TNF-α analysis. MSD cytokine analysis for TNF-α analysis was carried out using the proinflammatory panel 1 (mouse) kit (Meso Scale Discovery – MSD, Rockville USA) according to the manufacturer’s instruction. Plates were read on a MESO Quickplex SQ 120 imager (MSD, Rockville USA).

**Flow cytometry**

Using a FACSCalibur (BD Biosciences, San Jose CA) for flow cytometry, cell surface staining on freshly cultured splenocytes using four-colour dyes with the following mouse antibodies: fluorescein isothiocyanate-conjugated (FITC) CD 19, allophycocyanin-conjugated (APC) Ly6G, phycoerythrin-conjugated (PE) F4/80, Alexa Fluor 488 CD49b (Biolegend, London, UK), APC CD8a (eBioscience, Thermo Scientific, Ireland), APC CD3e, PE CD4 (BD Bioscience, Oxford, UK). Appropriate isotype-matched controls were used for each antibody. Cells (1 × 10^5^) were incubated with 1.0 μg of TruStain fcX^TM^ (Biolegend, London, UK) on ice for 5 - 10 min on ice to block Fc receptors. The appropriate antibodies were added and the cells incubated on ice in the dark for 30 min. Cells were washed twice by centrifugation at 400-600 x g for 5 min at room temperature with Staining buffer (Biolegend, London, UK) before flow cytometric analysis. A viability stain using 7-AAD Viability staining solution (Biolegend, London, UK) was also performed according to the manufacturer’s instruction to exclude dead cells. FACS Diva 6.2 software (BD Bioscience, Heidelberg, Germany) was used to analyse the data.

**Ethical approval, exclusion criteria and subject selection**

The Clinical Research Ethics Committee of the Cork Teaching Hospitals approved the human study. A total of 93 total subjects were recruited from the following cohorts: 31 elderly subjects from six long-term care facilities (long-stay, LS), 29 community-dwelling elderly subjects (community, EC), and 33 from a young healthy cohort (young-healthy, YH). The following exclusion criteria were used for subject selection: undergoing enteral (PEG) feeding, antibiotic usage in the preceding month, use of prebiotic, probiotic, fibre or symbiotic supplements in the preceding month, use of immunosuppressive or anti-inflammatory drugs or any drug affecting intestinal mobility, participants on warfarin, participation in another clinic or intervention study involving investigational drugs, including investigational prebiotic, probiotic or symbiotic preparations in the preceding 6 months, a history or evidence of gastrointestinal disorder, diagnosis of gastrointestinal cancer, a restricted dietary regimen, dysphagia, iron deficiency anaemia, malnourished subjects (determined by the initial MUST screen), participants suffering from hypochlorhydria and underlying chronic disease which affects their nutritional status and pregnant subjects.

## Peripheral blood mononuclear cell (PBMCs) isolation and flow cytometry

PBMCs were isolated from whole bloods sample volume 10 ml, collected in tubes containing ethylenediaminetetraacetic acid using the method by Boyüm [2]. After resuspending blood in phosphate buffered saline (PBS), cells were separated by density gradient centrifugation on histopaque. All reagents were from Sigma Aldrich, Ireland. The mononuclear cell layer was collected and washed twice in PBS. Cells were counted using a Neubaur cell counting chamber and resuspended staining buffer (eBioscience -Thermo Scientific Ireland) for CD4 and CD8 surface marker staining.

PBMCs (1 × 10^6^) were stained with the following human antibodies (Peridinin Chlorophyll Protein Complex (PerCP)-conjugated PerCP-eFluor^TM^710 CD4, PerCP-eFluor^TM^710 CD8a, eBioscience -Thermo Scientific, Ireland) and the isotype matched control as described in the protocol above.

## Cytokine and chemokine analysis

Coagulated blood was centrifuged for 10 min at full speed to separate cellular components and the serum harvested. Serum was aliquoted stored at -80°C and subsequently thawed on ice prior to use. All cytokines, chemokines were analysed according to the manufacturer’s instruction using the following MSD kits (MSD, Rockville, USA): V-PLEX Human IL17A, V-PLEX Proinflammatory Panel 1 Human, V-PLEX Human Eotaxin, Human I-TAC Tissue culture and Human CRP Kit.

## LPS endotoxin assay

Serum levels of LPS endotoxin was quantified using HEK-Blue^TM^LPS Detection Kit 2 (InvivoGen). In brief, HEK-Blue^TM^-4 cells were grown in growth medium for 48-72 h and then detached using a cell scraper when 90–95% confluence was reached, washed and 1×10^5^ cells transferred to each well of a 96-well microtitre plate (in growth medium). Serial dilutions of the sample and the HEK-Blue^TM^ Endotoxin Standard were prepared and added to the cell suspension in the 96 well plate. The plate was incubated for 24 h at 37°C in 5% CO_2_. QUANTI-Blue (180 μl) and 20μl of supernatant from the sample and standards were added to a new 96 well mircotitre plate. After incubation for 3 h at 37°C, the plates were read on a Microplate reader at 650 nm.

## DNA extraction, library preparation and 16S amplicon sequencing of human faecal samples

Genomic DNA was extracted from faecal samples (0.25 g) using the Repeat Bead Beating (RBB) method of Yu and Morrison [3] with the following modifications. Sterile zirconia beads (0.5 g) collections of one 3.0 mm bead, 0.1 g of 0.5 mm beads, and 0.3 g of 0.1 mm beads were used. Faecal samples were homogenised via bead beating for 90 seconds (Mini-Beadbeater™, BioSpec Products, Bartlesville, OK, USA), with the samples cooled on ice for 60 seconds before another 90 seconds bead beating. Pooled supernatants were incubated with 350 ml of 7.5 M ammonium acetate (Sigma). The extraction then proceeded as per the RBB extraction protocol. Genomic DNA was visualised on 1% agarose gel and quantified using the Nanodrop 1000 (Thermo Scientific, Ireland). Extracted genomic DNA was stored at -20°C until amplification. Bacterial primers used for PCR amplification targeting the V3-V4 hypervariable region of the 16S rRNA gene are V3 forward primer (TCGTCGGCAGCGTCAGATGTG-TATAAGAGACAGCCTACGGG-NGGCWGCAG) and V4 reverse primer (GTCTCGTGGGCTCGGAGATGTG-TATAAGAGACAGGACTACHVGGG-TATCTAATCC) [4]. Illumina overhang adapter sequences were appended to the 16S rRNA gene specific primer sequence. The PCR master mix comprised 0.2 μM forward and reverse 16S amplicon primers (EUROFINS, Ebersberg, Germany), 15 μl of 1X Phusion Taq High-Fidelity Mix (Thermo Scientific, Ireland), 10 ng DNA and nuclease free water to 30 μl. PCR program: 1. 98°C for 30s, 2. 98°C for 10s, 3. 55°C for 15s, 4. 72°C for 20s, 5. 72°C for 5 min. Steps 2-4 repeated 25 times. Amplicons were visually checked on 2% agarose gels and purified by magnetic purification using SPRI select beads (Beckman Coulter). The V3-V4 region of the 16S rRNA gene was then amplified using a limited cycle PCR and Nextera XT dual index barcodes (Illumina, Netherlands). The PCR master mix comprised 5μl forward and reverse Nextera primer respectively (Illumina, Netherlands), 25 μl of 1X Phusion Taq High-Fidelity Mix (Thermo Scientific, Ireland), 5μl PCR amplicon, and nuclease free water to 50 μl. The PCR program is the same as the aforementioned with the exception of 8 cycles repeated for steps 2-4. Amplicons were visually checked on 2% agarose gels, purified using SPRI select beads (Beckman Coulter) and quantified through fluorometric Qubit dsDNA HS Assay Kit (Thermo Scientific, Ireland). Equimolar amounts of DNA per amplicon were then pooled and sequenced at Teagasc, Fermoy, Ireland and GATC Biotech, Germany on an Illumina MiSeq using a 2x250 bp paired end sequencing run.

## DNA extraction and microbiota profiling of murine faecal samples

DNA was extracted from faecal samples using QIAamp DNA Stool Mini Kit (QIAGEN, UK) according to manufacturer’s protocol with the following modifications. Sterile zirconia beads (0.5 g) collections of one 3.0 mm bead, 0.1 g of 0.5 mm beads, and 0.3 g of 0.1 mm beads were used. Faecal samples were homogenised via bead beating for 30 seconds (Mini-Beadbeater™, BioSpec Products, Bartlesville, OK, USA), with the samples cooled on ice for 30 seconds before another 30 seconds bead beating, and then heated at 95°C for 5 min. The final elution volume was reduced to 50µL for murine faecal samples. The hypervariable 16S rRNA regions were amplified by PCR using the V4–V5 region primers 520F (AYTGGGYDTAAAGNG) and 926R (CCGTCAATTYYTTTRAGTTT) [5]. Amplicons were purified with Agencourt AMPure XP system (Beckman Coulter, UK) and sequenced on an Illumina MiSeq platform (GATC Biotech, Germany).

## Bioinformatic analysis

We applied a similar pipeline for 16S amplicon analysis for mouse and human gut microbiota, as follows: The Illumina MiSeq paired-end sequencing reads were joined using FLASh v1.2.8 [6]{Magoč, 2011 #1}{Magoč, 2011 #1} and filtered using split_libraries_fastq.py from QIIME v1.9.1 [7] with the default criteria. Quality-filtered reads were combined and processed with the USEARCH v6.1 [8]. Sequences were dereplicated, filtered by length, retaining sequences with lengths of 350–370 nt for V4-V5 region and 373–473 nt for V3-V4 region. After singleton removal, the remaining sequences for each amplicon type were clustered at 97% similarity into operational taxonomic units (OTUs) and filtered for chimeras against the GOLD reference database with UCHIME. All post-QIIME quality-filtered reads were then aligned to the filtered OTUs to generate an OTU table. Alpha and beta diversity metrics were calculated using QIIME. All quality-filtered reads were taxonomically classified with the RDP database trainset 14 [9] using mothur v1.36.1 and spingo v1.3 [10, 11]. Sequences with a confidence score below 50% were considered as unclassified.

## Statistical analysis

Differences in beta diversity were investigated using analysis of similarity (ANOSIM). Changes in alpha diversity were detected using linear mixed models (R package *nlme* [12]), with time point and treatment groups as fixed effects and subjects as a random effect. Analysis of variance (ANOVA) tests were used to investigate the effects of dosage difference on alpha diversity in linear mixed models. Differences of alpha diversity among cohorts and clinical data were compared by the Wilcoxon signed rank test for paired data, the Mann-Whitney U test for unpaired data or the Kruskal-Wallis test with Dunn's multiple comparison test to identify significant differences for three independent groups. Differential abundance at phylum and genus level among time points within treatment groups were identified using DESeq2 [13], controlling for subject identity across time-points, and HFD-index differences. *P-*values from DESeq2 were adjusted for multiple comparisons using Benjamini–Hochberg correction.

# References

1. Davies JM, MacSharry J, Shanahan F: Differential regulation of Toll-like receptor signalling in spleen and Peyer's patch dendritic cells. Immunology 2010, 131:438-448.

2. Boyum A: Isolation of leucocytes from human blood. Further observations. Methylcellulose, dextran, and ficoll as erythrocyteaggregating agents. Scand J Clin Lab Invest Suppl 1968, 97:31-50.

3. Yu Z, Morrison M: Improved extraction of PCR-quality community DNA from digesta and fecal samples. Biotechniques 2004, 36:808-812.

4. Klindworth A, Pruesse E, Schweer T, Peplies J, Quast C, Horn M, Glockner FO: Evaluation of general 16S ribosomal RNA gene PCR primers for classical and next-generation sequencing-based diversity studies. Nucleic Acids Res 2013, 41:e1.

5. Hill CJ, Brown JR, Lynch DB, Jeffery IB, Ryan CA, Ross RP, Stanton C, O'Toole PW: Effect of room temperature transport vials on DNA quality and phylogenetic composition of faecal microbiota of elderly adults and infants. Microbiome 2016, 4:19.

6. Magoč T, Salzberg SL: FLASH: fast length adjustment of short reads to improve genome assemblies. Bioinformatics 2011, 27:2957-2963.

7. Caporaso JG, Kuczynski J, Stombaugh J, Bittinger K, Bushman FD, Costello EK, Fierer N, Pena AG, Goodrich JK, Gordon JI, et al: QIIME allows analysis of high-throughput community sequencing data. Nat Methods 2010, 7:335-336.

8. Edgar RC: Search and clustering orders of magnitude faster than BLAST. Bioinformatics 2010, 26:2460-2461.

9. Cole JR, Wang Q, Fish JA, Chai B, McGarrell DM, Sun Y, Brown CT, Porras-Alfaro A, Kuske CR, Tiedje JM: Ribosomal Database Project: data and tools for high throughput rRNA analysis. Nucleic Acids Res 2014, 42:D633-642.

10. Schloss PD, Westcott SL, Ryabin T, Hall JR, Hartmann M, Hollister EB, Lesniewski RA, Oakley BB, Parks DH, Robinson CJ, et al: Introducing mothur: open-source, platform-independent, community-supported software for describing and comparing microbial communities. Appl Environ Microbiol 2009, 75:7537-7541.

11. Allard G, Ryan FJ, Jeffery IB, Claesson MJ: SPINGO: a rapid species-classifier for microbial amplicon sequences. BMC Bioinformatics 2015, 16:324.

12. Pinheiro J, Bates D, DebRoy S, Sarkar D and R Core Team. nlme: Linear and Nonlinear Mixed Effects Models. R package version 3.1-137 edition; 2018.

13. Love MI, Huber W, Anders S: Moderated estimation of fold change and dispersion for RNA-seq data with DESeq2. Genome Biol 2014, 15:550.
